# AI identifies potent inducers of breast cancer stem cell differentiation based on adversarial learning from gene expression data

**DOI:** 10.1093/bib/bbae207

**Published:** 2024-05-02

**Authors:** Zhongxiao Li, Antonella Napolitano, Monica Fedele, Xin Gao, Francesco Napolitano

**Affiliations:** Computer Science Program, Computer, Electrical and Mathematical Sciences and Engineering Division, King Abdullah University of Science and Technology (KAUST), Thuwal, 23955, Saudi Arabia; Computational Bioscience Research Center, King Abdullah University of Science and Technology, Thuwal, 23955, Saudi Arabia; Institute of Experimental Endocrinology and Oncology “G. Salvatore” (IEOS), National Research Council (CNR), Via De Amicis, 95 - 80131 Napoli, Italy; Institute of Experimental Endocrinology and Oncology “G. Salvatore” (IEOS), National Research Council (CNR), Via De Amicis, 95 - 80131 Napoli, Italy; Computer Science Program, Computer, Electrical and Mathematical Sciences and Engineering Division, King Abdullah University of Science and Technology (KAUST), Thuwal, 23955, Saudi Arabia; Computational Bioscience Research Center, King Abdullah University of Science and Technology, Thuwal, 23955, Saudi Arabia; Computational Bioscience Research Center, King Abdullah University of Science and Technology, Thuwal, 23955, Saudi Arabia; Department of Science and Technology, University of Sannio, Via dei Mulini 74, 82100 Benevento, Italy

**Keywords:** artificial intelligence, domain adaptation, transcriptomics, drug repurposing, cancer stem cells, breast cancer

## Abstract

Cancer stem cells (CSCs) are a subpopulation of cancer cells within tumors that exhibit stem-like properties and represent a potentially effective therapeutic target toward long-term remission by means of differentiation induction. By leveraging an artificial intelligence approach solely based on transcriptomics data, this study scored a large library of small molecules based on their predicted ability to induce differentiation in stem-like cells. In particular, a deep neural network model was trained using publicly available single-cell RNA-Seq data obtained from untreated human-induced pluripotent stem cells at various differentiation stages and subsequently utilized to screen drug-induced gene expression profiles from the Library of Integrated Network-based Cellular Signatures (LINCS) database. The challenge of adapting such different data domains was tackled by devising an adversarial learning approach that was able to effectively identify and remove domain-specific bias during the training phase. Experimental validation in MDA-MB-231 and MCF7 cells demonstrated the efficacy of five out of six tested molecules among those scored highest by the model. In particular, the efficacy of triptolide, OTS-167, quinacrine, granisetron and A-443654 offer a potential avenue for targeted therapies against breast CSCs.

## INTRODUCTION

Cancer stem cells (CSCs) are a subpopulation of cancer cells within tumors that exhibit stem-like properties, including the ability to undergo self-renewal and asymmetric division giving rise to copies of themselves and the mature progeny of non-stem cells through differentiation. CSCs may mediate tumor metastasis and relapse, thus representing a potentially effective therapeutic target toward long-term remission by means of differentiation induction [1]. It has been noted that even partial success of differentiation therapy could improve the prognosis of most patients by decades [2]. Differentiation therapy represents a paradigm case in acute myeloid leukemia (AML), where terminal differentiation of CSCs has been shown to produce significant clinical benefits [3]. Although it has been proposed that such benefits in AML are not exclusively due to differentiation of CSCs, differentiation therapy still holds tremendous therapeutic hope, also for solid tumors [4–7]. In fact, CSCs have been identified in a broad spectrum of solid tumors [8], including breast cancer (BC) [9]. It has also been demonstrated that despite the fact that prolonged *in vitro* culturing is thought to result in loss of crucial stemness properties, established BC cell lines possess a small fraction of self-renewing tumorigenic cells with the capacity to differentiate into phenotypically diverse progeny. BC stem cell (BCSC) content varies greatly among BC cell lines and breast carcinomas [10, 11]. Triple-negative BCs (TNBCs) contain large numbers of BCSCs, while luminal breast tumors have lower stem cell contents [12, 13]. Consistently, the MCF7 luminal BC cell line has a low percentage (0.7–1.4%) of BCSCs, while the MDA-MB-231 TNBC cell line exhibits low or null CD24 expression and high percentage (more than 90%) of CD44+ cells [14]. BCSCs are able to undergo self-renewal, give rise to phenotypically diverse progeny and survive chemotherapy, thereby constituting an excellent model for CSCs [14]. Moreover, supporting evidence for a hierarchical CSC-based model of metastasis initiation has been provided through single-cell analysis of human metastatic BC cells [15]. Stemness properties were also identified by analyzing transcriptomic data of BC cells from patients [16].

Given the potential of differentiation therapy and the evidence of CSCs in a broad spectrum of tumors, searching for small molecules that can target CSCs is an active area of research. For example, histone deacetylase inhibitors have been investigated for differentiation therapy in AML on the basis of their epigenetic effects [17]. In general, multiple methodologies have been proposed that leverage small-molecule treatment to augment cell conversion [18]. These encompass numerous applications for cell reprogramming or trans-differentiation, including but not limited to, neurons [19], endothelial cells [20], pancreatic-like cells [21], cardiomyocytes [22], hepatocytes [23] and other types of cells [24–26]. However, a relatively poor understanding of differentiation mechanisms [2] has prevented a systematic rational approach to the discovery of novel effective molecules. It is therefore unsurprising that, in the context of CSCs targeting, one of the major studies involved a high-throughput screening (HCS) approach, through which the ability of salinomycin in selectively killing BCSCs was discovered [27]. All these investigations underscore the potential of drug-enhanced cell type conversion, although they often require extensive experimentation and/or prior understanding of biological targets, making the screening of large small-molecule libraries a remarkably challenging task. In contrast, computational methods can provide practical shortcuts to identify small sets of promising candidates for subsequent validations. While a large number of target-aware computational methods for clinical applications have been proposed [28], including integrated approaches exploiting heterogeneous data types [29–31], we have recently introduced a general target-agnostic method for prioritizing small molecules in diverse cell conversion scenarios solely based on drug-induced transcriptional data, termed ‘DECCODE’ [32]. The method’s efficacy was validated in a cell reprogramming protocol, showing promising results as a tool for differentiation studies as well. In particular, it was used to screen the LINCS [33] database to search for stemness signatures among ~20 000 drug-induced gene expression profiles (GEPs).

**Figure 1:**
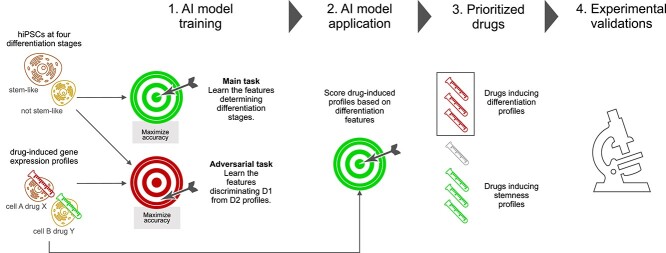
Overview of the study. Single-cell GEPs of hiPSCs at various differentiation stages and drug-induced GEPs were fed to an adversarial learning model, which simultaneously learned differentiation features to be used in subsequent predictions (main task) and dataset-specific features to be avoided (adversarial task). The trained model was then used to score all the drug-induced profiles. A selection of six drugs among the top-scoring ones was experimentally validated.

While the DECCODE approach is based on classical statistics to match a single target profile, a large number of samples representing the desired transcriptional profile would allow for the application of more advanced machine learning models, which are likely to yield improved accuracy. This advancement in accuracy, coupled with the potential benefits of differentiation therapy in BC, underpins the main motivation for the present study (overviewed in Figure 1). Exploiting publicly available single-cell RNA-Seq (scRNA-Seq) data from human-induced pluripotent stem cells (hiPSCs) labeled according to four differentiation stages, we devised an artificial intelligence (AI) approach to learn the corresponding expression patterns and subsequently prioritize drugs based on their ability to induce similar features. This approach allows for completely data-driven drug-prioritization, not relying on known specific targets or in general any prior knowledge about the biological mechanisms involved. On the other hand, it poses the significant challenge of training an artificial neural network from an scRNA-Seq dataset of untreated cells and using it to evaluate drug-induced profiles from the LINCS L1000-based collection, i.e. two completely different platforms and cellular contexts. We tackled the problem by developing ‘DREDDA’ (‘Drug Repositioning through Expression Data Domain Adaptation’), a domain-adaptive adversarial architecture that was able to learn and remove most of the domain-specific information from the two datasets while simultaneously solving the main task of identifying differentiation patterns. In particular, the technique allowed the model to learn domain-specific features (adversarial task) during the training phase and simultaneously avoid their use during differentiation stage classification (main task). Domain adaptation was first widely explored in visual recognition tasks, where it aims to apply visual recognition models trained in one domain (e.g. photos) to another domain (e.g. paintings) [34–36]. Along the same principles, DREDDA was designed to learn cell differentiation patterns from the scRNA-Seq dataset and use such acquired knowledge to predict the differentiation-induction ability of each drug from the LINCS collection. Finally, six of the most interesting hits from the resulting drug prioritization were experimentally validated, demonstrating the efficacy of five of them in reducing CSCs in MCF7 and MDA-MB-231 cell lines.

## RESULTS

### Model development

With the aim of identifying BCSC differentiation-inducing molecules, we designed an AI approach completely based on transcriptional data (see Supplemental Methods section). The fundamental idea was to use a machine learning model in two steps: (1) learn transcriptional patterns that can discriminate stem cells from differentiated cells and (2) use the trained model to identify small molecules inducing similar patterns in treated cells. Toward this aim, we correspondingly exploited two different datasets: (1) an scRNA-seq dataset of hiPSCs including information about the differentiation stage of each cell and (2) a database of drug-induced transcriptional profiles obtained after the treatment of different cell lines. In particular, the scRNA-seq dataset we selected includes 18 787 hiPSCs obtained from WTC-CRISPRi [37] cells. After sequencing, each cell was assigned one of four differentiation stages based on unsupervised clustering and biomarker analysis. As for the second dataset, we used drug-induced transcriptional profiles obtained from the LINCS dataset available at the Gene Expression Omnibus (GEO: GSE70138), including 107 404 differential GEPs corresponding to the transcriptional responses of 41 cell lines to 1768 different small molecules spanning different concentrations and time points [33].

**Figure 2:**
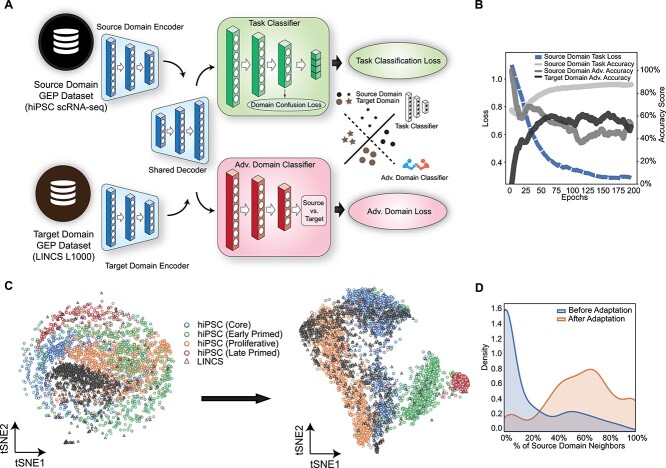
Model development. (**A**) The DREDDA model architecture includes one encoder for each dataset and a shared decoder; the resulting profiles from the source domain are sent to the main task classifier (positively weighted in the overall loss function), while both source and target domain profiles are sent to the adversarial classifier (negatively weighted). (**B**) During training, the main task accuracy increases, while the adversarial task accuracy decreases. (**C**) Comparison between the embedding before (left) and after (right) domain adaptation shows that cells at the various differentiation stages tend to cluster together more, while LINCS drug-induced profiles tend to spread across the source domain. (**D**) The neighborhood of untreated cell profiles tends to be more enriched for LINCS profiles after domain adaptation (curve peaking to the right) as compared to before (curve peaking to the left).

Since the model needs to be trained with the first dataset and provide predictions for the second one, the main challenge in its development was to effectively adapt the two domains, both of which are affected by biological and technical biases. The main source of biological bias came from the different cell types involved in both datasets. Although the cellular context represents an obviously relevant biological variable, it also acts as a severe limiting factor to the applicability of large drug-induced gene expression datasets. For this reason, methods treating cell type variability as biological bias have been proposed with the aim of maximizing drug prioritization performances from the available data [38]. The rationale is that the treatment effects observed in the available transcriptional data even after correcting for cell types should not be bound to a specific cellular context. Concerning technical biases, the two datasets were produced with remarkably different technologies, i.e. scRNA-Seq and L1000, the latter being specifically designed within the LINCS project. In order to reduce such sources of misleading signals, we devised an adversarial domain adaptation approach (Figure 2a and Supplemental Methods section), in which a single deep learning model was trained to solve two competing tasks: (1) the main task, i.e. identifying the differentiation stage of each cell from the hiPSCs dataset and (2) the adversarial task, i.e. to discriminate between hiPSCs profiles and LINCS profiles (regardless of the treated cell line). In particular, the model was trained to maximize the performance of the main task and simultaneously minimize the performance of the second task. In this way, the extracted transcriptional features allowed the prediction of differentiation stages without relying on domain-specific information. During the training phase, the hiPSC dataset alone was used for the main task, while both datasets were used for the adversarial task. In particular, the training phase of DREDDA aimed for a steady increase of the main task classification performance and a steady decrease of the adversarial domain classification performance (Figure 2B). Indeed, the main task on the hiPSC dataset achieved 86.7% accuracy at the end of the training, significantly improving from the initial low performance. On the other hand, the adversarial task accuracy started at 100%, highlighting a severe dataset-dependent bias, but reached a ~50% performance by the end of the training (Figure 2B), indicating near- complete inability to distinguish between hiPSC and LINCS profiles. In other words, the information extracted by the model was sufficient to perform the main task, although largely irrelevant to the adversarial task. The internal representation of the data defined by the model after domain adaptation is visualized in Figure 2C together with a representation of the original data space. By comparing the two representations, it is evident how the clusters of cells belonging to each of the four differentiation stages appear significantly more separated after domain adaptation. On the other hand, the LINCS profiles, which mostly clustered together before adaptation, appear widely spread after adaptation, making them hardly separable from hiPSC profiles. We also quantified this effect by counting the percentage of hiPSC profiles falling in the 30 nearest neighbors of each LINCS profile before and after adaptation, showing a dramatic shift in the corresponding distributions (Figure 2D).

### Top hits validation and characterization

**Figure 3:**
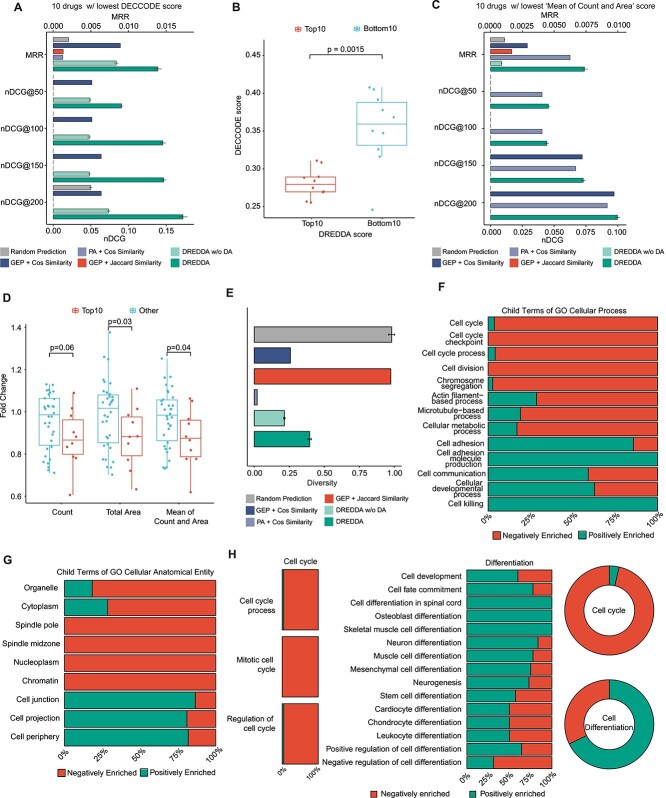
Validation and characterization of the top hits. (**A**) The performance of DREDDA and the other five tested methods as measured by the MRR and the nDCG at four different thresholds (@50, 100, 150, 200) of the bottom 10 drugs with the lowest DECCODE scores (see Supplemental Methods for the detailed descriptions). Error bars displayed for DREDDA, DREDDA w/o DA and Random Prediction based on five independent runs. (**B**) Top (bottom) drugs as prioritized by DREDDA have low (high) DECCODE scores, which predict stemness features (**C**) Similar to (**A**) but using the top 10 drugs which resulted in the highest colony area and counts. (**D**) Drugs previously tested for inducing stemness tend to be ranked lower by DREDDA based on experimental evidence including stem cells’ colony count and size. (**E**) The classification diversity of the LINCS profiles by DREDDA and the five other comparing methods into the four states of hiPSCs (**F**, **G**) Summary of the positive and negative enrichments for pathways among the top levels of the ‘Biological Process’ and ‘Cellular Component’ Gene Ontology categories that are significantly dysregulated by the top 30 drugs. (**H**) Same analysis as in (**F**, **G**), but focused on the ‘Cell cycle’ and ‘Differentiation’ levels in the ‘Biological Process’ category.

After training, the model was finally used to perform the main task on each of the LINCS profiles and thus predict the effectiveness of the corresponding treatment to induce the transcriptional features learned from the hiPSC dataset. In particular, we used the scores assigned by the model as a prioritization measure to rank all LINCS profiles. In order to validate the prioritization based on prior knowledge, we collected DECCODE scores for all the drugs in the list of DREDDA predictions. DECCODE is a measure of stemness based on biomarker identification from time series gene expression data obtained through *ad hoc* cell reprogramming experiments that we defined and validated in a previous study [32]. Given its meaning, we expected to observe a tendency of DECCODE scores to induce opposite predictions as compared to DREDDA scores. To verify this tendency, we applied two commonly used information retrieval (IR) metrics: (1) mean reciprocal rank (MRR) and (2) normalized discounted cumulative gain (nDCG, computed at four different cutoffs: 50, 100, 150, 200). In particular, we first ranked the drugs in the LINCS database according to the scores assigned by the DREDDA model and then computed MRR and nDCG for the set of 10 drugs with the lowest DECCODE scores (see Supplemental Methods). Moreover, we repeated the same analysis after ranking the drugs according to five additional prediction methods: (1) random, (2) average cosine similarity of LINCS GEPs to the GEP signatures of the hiPSC clusters (‘GEP + Cos Similarity’), (3) analogous approach using the Jaccard similarity (‘GEP + Jaccard Similarity’), (4) average cosine similarity of pathway activations (PAs) [39], which compares profiles at the pathway level (‘PA + Cosine Similarity’) and (5) DREDDA without domain adaptation (‘DREDDA w/o DA’) (see Supplemental Methods). DREDDA consistently and significantly outperformed the other methods. The remarkable improvement after including domain adaptation underlines the fundamental importance of this harmonization step in integrating highly heterogeneous transcriptomics data (Figure 3A). Consistent with previous literature [39], such effect of the harmonization is also observed during the conversion of GEPs to PAs as it strongly boosted the performance of cosine similarity. Additionally, we also observed statistically significant low (high) DECCODE scores in the top- (bottom-) 10 drugs (Figure 3B), which appear coherent with differentiation (stemness) features. We also observed a general negative correlation between the DREDDA score and the DECCODE score (Figure S1).

Apart from the validation of DREDDA’s prediction with the previous computational methods, we also specifically tested its consistency with previously published experimental results on a collection of 45 drugs, including 25 with high DECCODE scores and 20 with low DECCODE scores [32]. Briefly, these drugs were tested for pluripotency induction in human inducible fibroblast-like cells by means of colony formation assays. Following each treatment, the count and size (% of plate area covered) of the forming colonies were used to measure the efficacy of pluripotency induction. Additionally, a combined measure was obtained by calculating the average percent increase in both colony count and size relative to untreated cells. For the present study, we selected the 10 drugs with the smallest combined measure and computed the IR metrics for all the methods as previously described. Also in this case, DREDDA outperformed the other methods in terms of MRR and nDCG (Figure 3C). In contrast, none of the 10 drugs were selected among the top 50 or 100 drugs as ranked by three of alternative methods (random prediction, GEP + Cos similarity, GEP + Jaccard similarity), resulting in the corresponding null scores. We also sorted the list of 45 drugs according to their DREDDA scores and observed that the 10 drugs with the highest DREDDA scores generally induced low colony count and colony-covered area in the DECCODE-related experiments, thus providing one of the first experimental evidence of the effects induced by such drugs on cell stemness (Figure 3D). Finally, we evaluated an additional important parameter for all the methods, i.e. prediction diversity (Figure 3E and Supplemental Methods), which may explain the higher performance of DREDDA in terms of lower prediction bias.

Among the top 30 drugs prioritized by DREDDA (Table S1), many molecules belong to chemotherapeutic agents in the class of kinase inhibitors, including CDK inhibitors, MELK inhibitors and JNK inhibitors. Other molecules specifically target DNA replication, including topoisomerase inhibitors and pyrimidine synthesis inhibitors. In order to further explore their common molecular features, we took advantage of the corresponding LINCS profiles. We first extracted the 30 most dysregulated genes from each of the top 30 profiles. As expected from the inhibitory nature of many drugs in the set, the dysregulated genes appeared to be mostly down-regulated (Figure S2). Specifically, the same 19 genes were commonly down-regulated by more than 10 drugs, but only the 7 same genes were commonly up-regulated by more than 10 drugs (Figure S2). Many of the 19 down-regulated genes are related to the cell cycle. For example, the expression of the proliferating cell nuclear antigen (PCNA), essential for DNA replication, appeared reduced by 22 drugs in the list, while Cyclin B2 (CCNB2) appeared down-regulated by 23 drugs (Table S2). This was better assessed by an enrichment analysis performed through the DAVID tool [40], which not only confirmed a clear enrichment of cell-cycle-related pathways but also highlighted the presence of two differentiation related pathways (Table S3). However, in order to directly and systematically investigate the common pathways affected by the top 30 drugs, we resorted to a specific tool, i.e. the Drug Set Enrichment Analysis (DSEA) [41], using the ‘Biological Process’ and ‘Cellular component’ categories of the Gene Ontology (GO) collection (Tables S4 and S5). The most significant resulting pathways with a negative score included many that are associated with the cell cycle process (such as cell cycle G2-M phase transition, positive regulation of cyclin-dependent protein kinase activity and telomerase RNA localization) and structures involved in it (including nuclear envelope, spindle pole and centrosome). On the other hand, the most significant pathways with a positive enrichment score mostly concerned cell communication (e.g. regulation of calcium ion transmembrane transport; regulation of hormone levels; organic anion transport) or differentiation (pattern specification process; regionalization; photoreceptor cell differentiation). Next, in order to obtain a more high-level overview of the most recurrent cellular activities impacted by the drug set, we systematically investigated the up- and down-regulation of pathways falling within larger families of biological processes and cellular components. In particular, we quantified how many negatively and positively DSEA-enriched pathways fell below each one of the top terms in the GO hierarchy (Figure 3F). Notably, most pathways in the families of cell cycle (i.e. cell cycle, cell cycle checkpoint and cell cycle process) and cell division (i.e. cell division, chromosome segregation, actin filament–based process and cellular metabolic process) were negatively enriched, suggesting a general inhibition of the cell cycle progression under the treatment of the top 30 drugs. In contrast, most pathways within families that are possibly related to cell differentiation (i.e. cell adhesion, cell communication and cellular developmental process) were positively enriched. Consistently, the same analysis on top-level pathways in the Cellular Component category showed that most cell cycle–related cellular structures were negatively enriched (e.g. spindle pole, nucleoplasm and chromatin), while those possibly related with differentiation through cell communication were positively enriched (to cell junction, cell projection and cell periphery) (Figure 3G). All such results were obtained by blindly investigating pathways and families of pathways without using any prior information. However, given the known desired effects that the drugs were prioritized for by DREDDA, we further investigated the enrichment of pathways below the cell cycle and cell differentiation levels in the GO hierarchy (Figure 3H). All of the three levels below cell cycle (i.e. cell cycle process; mitotic cell cycle; regulation of cell cycle) were highly enriched by negatively regulated pathways. On the other hand, most levels below the cell differentiation term appeared positively enriched.

### *In vitro* biological evaluation: effects of the molecules on general BC cell viability

Computational results were validated through *in vitro* experiments using the MCF7 (luminal triple-positive BC) and MDA-MB-231 (mesenchymal-like triple-negative BC) cell lines, chosen as models of BC with low and high percentages of CSCs, respectively [42]. Six small molecules (Table 1), out of the top 50, were selected based on their availability and interest. In particular, in order to obtain a small but diverse set of candidates, two drugs were selected solely based on their ranks (first and second in the prioritization), two other drugs for being already approved in oncological applications, one drug for being approved in an unrelated context and one small molecule with no approved clinical application (see Discussion for further considerations). From a preliminary exploration concerning their common mode of action, we observed that quinacrine, triptolide and A-443654 target different components of the AKT pathway (Figure S3). On the other hand, triptolide and quinacrine both target the NF-κB pathway, albeit through different mechanisms [43, 44]. OTS-167 and A-443654 both affect cell cycle regulation, with OTS-167 targeting PLK1 and A-443654 targeting AKT, which influences cell cycle progression [45, 46]. Triptolide, OTS-167, A-443654 and quinacrine all have anti-neoplastic properties, although they target different pathways involved in cancer cell growth and survival. The six molecules were first tested for cell viability using increasing drug concentrations to establish the IC50 (Figure S4). According to the MTT findings, triptolide and OTS-167 were highly cytotoxic in both cell lines with IC50 at nanomolar concentrations. A-443654 showed similar IC50 as compared to OTS-167 only on MCF7 cells, while it was less effective on the more staminal and therefore chemo-resistant MDA-MB-231 cell line. Granisetron and leflunomide were better tolerated by both cell lines, resulting in IC50 at micromolar concentrations. Finally, quinacrine showed an intermediate IC50 in the low micromolar for both cell lines. Based on these findings we chose the working concentrations to be used for each molecule in the following assays targeting CSCs. Two significantly different effective dosages were used for all the molecules, as detailed in Table S6.

**Table 1:** Small molecules selected for experimental validation from the top hits in the prioritization list

Drug	2D structure	Targets	Clinical trials and approvals
Triptolide	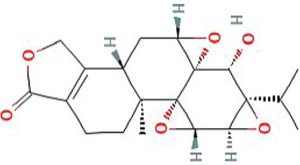	EGFR, HSP70 heat-shock proteins, NFKB1, NFKB2, RELA, RELB, REL, Myc, γ-secretase complex	Autoimmune diabetes, Autosomal Dominant Polycystic Disease (Phase 3) Psoriasis (Approved)
OTS-167	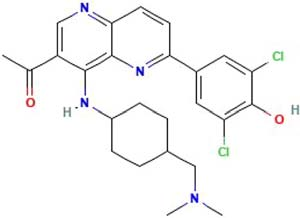	MELK	Relapsed/Refractory Locally Advanced or Metastatic Breast Cancer and Triple Negative Breast Cancer (Phase 1) Chronic Myelogenous Leukemia, Myelodysplastic Syndromes, Acute Lymphoblastic Leukemia, Acute Myeloid Leukemia (Phase 2)
A-443654	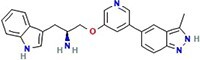	AKT1 AKT2 AKT3	N/A
Leflunomide	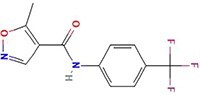	Malaria DHOdehase	PTEN-null Advanced Solid Malignancies (Phase 1) Arthritis (Approved) Multiple sclerosis (Approved)
Granisetron	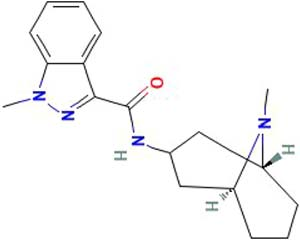	5HT3R	Nausea and vomiting (Approved)
Quinacrine	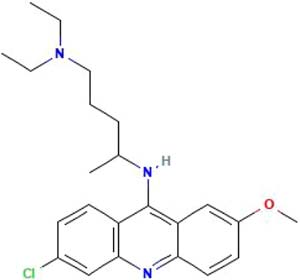	PLA2G1B p53 NFkB	Advanced Renal Cell Carcinoma, Prostate cancer (Phase 2) Giardiasis, Leishmaniasis, Malaria, Systemic Lupus Erythematosus (Approved)

### Validation of molecule efficacy on BCSCs

**Figure 4:**
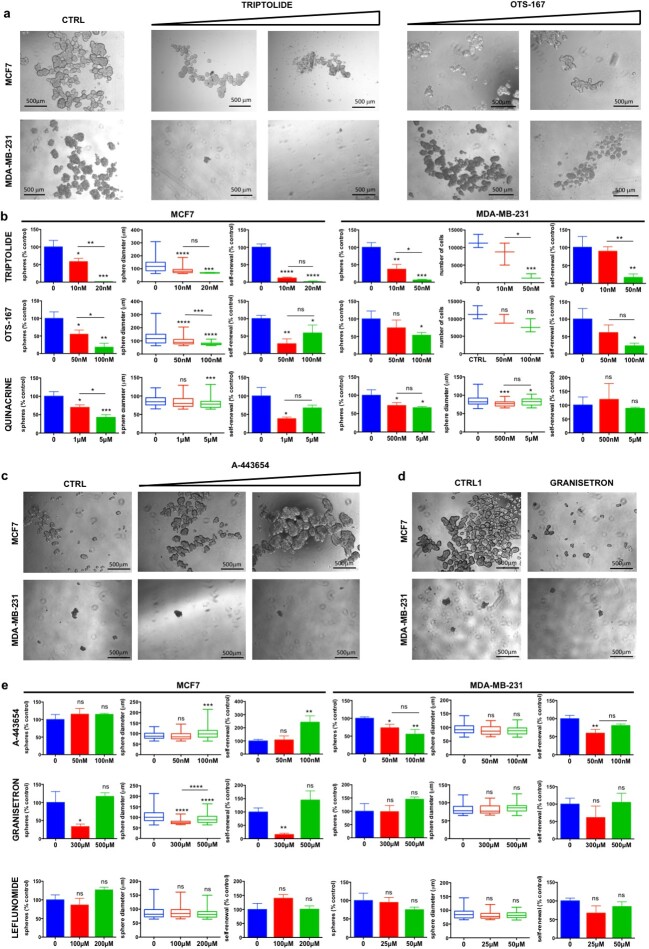
Mammosphere assay in drug-treated BC cells. (**A**) Representative images of MCF7 and MDA-MB-231 cells pre-treated for 24 h with increasing doses of the indicated molecules and then cultured for 7 days as mammospheres in stem cell medium after the washing out of the drug. (**B**) Average number of mammospheres, their diameter and self-renewal capacity in three independent experiments. For MDA-MB-231 treated with triptolide and OTS-167, the number of single cells composing the mammospheres, as a measure of their growth, is reported instead of the mammosphere diameter. (**C**, **D**) Representative images of MCF7 and MDA-MB-231 cells pre-treated for 24 h with increasing doses of each molecule and then cultured for 7 days as mammospheres in stem cell medium after the washing out of the drug. (**D**) For granisetron, only the lower dose (300 μM) and its relative control (CTRL1) were shown. (**E**) Average number of mammospheres, their diameter and self-renewal capacity in three independent experiments. CTRL = DMSO 0.1%; CTRL1 = DMSO 0.6%; ^*^, *P* < 0.05; ^*^^*^, *P* < 0.01; ^*^^*^^*^, *P* < 0.001; ^*^^*^^*^^*^, *P* < 0.0001; ns, not significant.

To evaluate the effects of each drug on BCSCs, we first treated adherent cells for 24 h; then, we washed out the drug and seeded the surviving cells in stem cell medium on ultra-low attachment plates to let only BCSCs growing as mammospheres. The analysis of mammosphere-forming efficiency (MFE), growth ability and self-renewal showed that three drugs, triptolide, OTS-167 and quinacrine, effectively suppressed the growth of BCSCs in both cell lines (Figure 4A and B). In more detail, triptolide decreased the MFE of both MDA-MB-231 and MCF7 cells in a dose-dependent manner. It also reduced mammosphere growth ability and self-renewal for both cell lines by nearly 80% and 90% at the highest dose. OTS-167 inhibited MFE and self-renewal activity of MDA-MB-231 cells in a dose-dependent manner, while their growth ability was significantly inhibited only at the highest dose. OTS-167 also decreased MFE and growth of MCF7 cells in a dose-dependent manner, while self-renewal was highly reduced at both doses without significant differences between them. Quinacrine showed a significant effect on the reduction of MFE and self-renewal (dose-dependent only for MFE) of MCF7 cells, while its effect on MDA-MB-231 cells was only a significant reduction of mammosphere growing ability and a trend for a reduced MFE (Figure 4B). Other two drugs, granisetron and A-443654, inhibited MFE, mammosphere growth and self-renewal of either MCF7 or MDA-MB-231, respectively (Figure 4C–E). For granisetron, only the lower dose (300 μM) showed a significant effect. Finally, leflunomide did not show any significant effect on BCSC availability and growth of both cell lines (Figure 4E).

### Induction of BCSC differentiation

**Figure 5:**
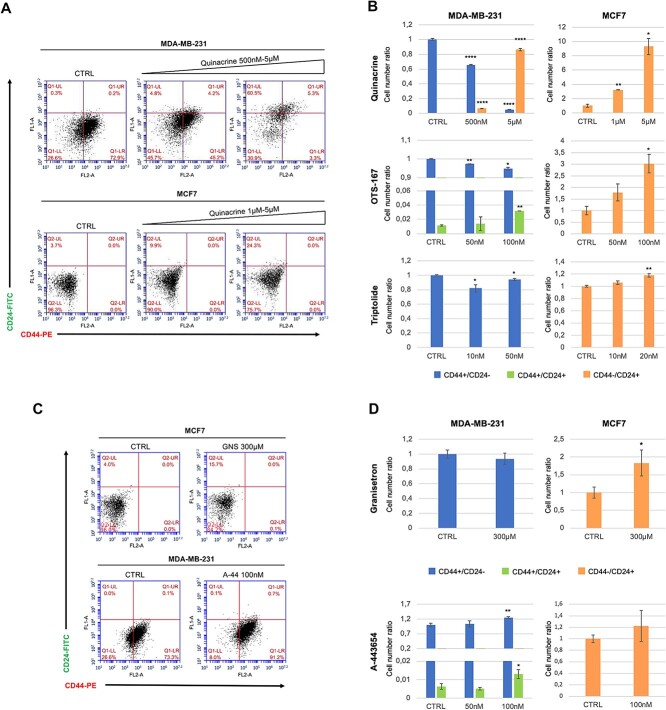
FACS profiling of CD44 and CD24 expression in MDA-MB-231 and MCF7 cells treated with quinacrine, triptolide, OTS-167, granisetron and A-443654. (**A**) Representative dot plots for quinacrine-treated cells. (**B**) The mean values +/− SE of the CD44+/CD24- (blue bars), CD44+/CD24+ (green bars) and CD44-/CD24+ (orange bars) subpopulations were reported as a ratio relative to control (CTRL: DMSO 0.1%) for all treatments. (**C**) Representative dot plots of granisetron-treated MCF7 and A-443654-treated MDA-MB-231 cells. GNS, granisetron; A-44, A-443654. (**D**) The mean values +/− SE of the CD44+/CD24- (blue bars), CD44+/CD24+ (green bars) and CD44-/CD24+ (orange bars) subpopulations were reported as a ratio relative to control (CTRL: DMSO 0.1% for A-443654; DMSO 0.6% for granisetron) for all treatments. ^*^, *P* < 0.05; ^*^^*^, *P* < 0.01; ^*^^*^^*^^*^, *P* < 0.0001.

BCSCs are classically defined by CD44 (Cluster of Differentiation antigen-44) positive and low or absent levels of CD24 (Cluster of Differentiation antigen-24) expression (CD44^+^/CD24^−/low^) on their surface [47] and recent clinical evidence has established that tumorigenic BC cells with high expression of CD44 and low expression of CD24 are resistant to chemotherapy [48]. To evaluate whether the effect of the drugs on the availability and growth capacity of BCSCs was due to the induction of their differentiation, as predicted by the AI algorithm, we analyzed CD44 and CD24 expression by fluorescence-activating cell sorting (FACS), on adherent MDA-MB-231 and MCF7 cells treated for 24 h with each of the molecules that showed significant effects in the mammosphere assay. Quinacrine was found to be the most effective drug in inducing BCSC differentiation in both MCF7 and MDA-MB-231 cells, as assessed by the large dose-dependent decrease and simultaneous increase of the CD44^+^/CD24^−^ and CD44^−^/CD24^+^ subpopulations, respectively, in the MDA-MB-231 cells and significant dose-dependent increase of the CD44-/CD24+ subpopulation in the MCF7 cells (Figure 5A and B). However, triptolide and OTS-167 also showed significant differentiating effects on both MCF7 and MDA-MB-231 cells (Figure 5B), while granisetron and A-443654 showed significant differentiating effects only on MCF7 or MDA-MB-231, respectively (Figure 5C and D), consistently with the mammosphere assay. These results confirm that the effects of the selected drugs on the availability, growth capacity and self-renewal of BCSCs are due to the induction of their differentiation.

## DISCUSSION

BC is a complex disease characterized by cellular heterogeneity among which the presence of CSCs has been identified as a key factor contributing to tumor initiation, progression and therapy resistance, thereby indicating an important therapeutic target. In this study, an AI approach was employed to identify potential differentiating agents targeting BCSCs. The utilization of AI offered a powerful tool for screening a large library of compounds and identifying molecules with desired properties. Previous studies have demonstrated that drug-induced gene expression data, regardless of its known technical limitations and biological context dependency, can be effectively used to prioritize molecules facilitating cell-type conversion based on a specific target expression profile. In this study, for the first time we showed how the same idea could be extended to an even more agnostic case, in which the target differential profile itself is not defined a priori, but learned from data, exploiting domain adaptation across GEPs of treated and untreated cells for improved comparability. In particular, this was made possible by a machine learning approach that automatically extracted relevant transcriptional features from scRNA-seq data. Indeed, from a computational perspective, single-cell transcriptomics proved to be an effective platform to obtain sizable datasets that are suitable for the training and testing of machine learning algorithms across diverse domains, despite the severe biases involved. With the aim of ameliorating such bias, possibly relevant cell-specific features were likely removed during the training phase, which may represent the major drawback of this approach. Nonetheless, this strategy is necessary to deal with the limited availability of consistent drug-induced GEP datasets, another significant challenge for this type of data-driven discovery algorithms.

Following the AI-based screening, the study experimentally validated the efficacy of five out of six selected molecules, namely, triptolide, OTS-167, quinacrine, granisetron and A-443654, in targeting BCSCs by inducing them to differentiate. Two commonly studied BC cell lines, MCF7 and MDA-MB-231, were used to assess the impact of these compounds on BCSCs, showing effective suppression of mammosphere-forming efficiency, growth and self-renewal. The differentiation induction was confirmed by an altered protein expression associated with stemness and differentiation. Indeed, the CD44+/CD24− subpopulation was reduced in MDA-MB-231, while CD24+ cells were increased in both MDA-MB-231 and MCF7 cells.

In several previous studies, triptolide, a natural compound (diterpenoid tri-epoxide) derived from the Chinese herb *Tripterygium wilfordii*, has been found to exhibit potent anti-cancer properties, including anti-proliferative, anti-metastatic and pro-apoptotic effects in various cancer types [49–52]. Some of these studies explored the potential of triptolide in targeting BCSCs, showing it inhibits multiple signaling pathways involved in self-renewal and maintenance of BCSCs, including c-Myc, Wnt/β-catenin and Notch pathways [53–55]. Consistent with our results, Li *et al*. [56] demonstrated that triptolide inhibited self-renewal and induced a more differentiated phenotype in BCSCs, leading to reduced tumor growth and metastasis.

Similarly, there have been studies investigating the role of quinacrine, a well-known antimalarial drug, in targeting CSCs [57]. Specifically, quinacrine treatment effectively inhibited cell proliferation, migration, invasion and representative metastasis markers of BCSCs [58, 59]. However, no studies have so far shown a direct role of this agent on CSC properties in BC or other tumor models.

OTS-167, also known as OTSSP167, is an orally available MELK (Maternal embryonic leucine zipper kinase) inhibitor that is currently in phase I/II clinical trials for various tumors [60]. MELK induces carcinogenesis effects and is tightly associated with extended survival and accelerated proliferation of CSCs in various tumors, including glioblastoma and BC [61]. Consistently, MELK inhibition by OTS-167 treatment significantly suppresses the proliferation and neurosphere formation in glioblastoma stem cells, in which MELK expression is enriched [62]. However, there is limited research specifically focused on the role of OTS-167 in BCSCs. Only Chung *et al*. [63], in their pioneer study on the development of this compound, investigated its direct impact on BCSCs, demonstrating its efficacy in suppressing mammosphere formation and tumor growth in xenograft studies. Here, we confirmed its efficacy in reducing BCSC availability, growth and self-renewal by mammosphere assays, also showing that it induces their differentiation.

Granisetron, a selective serotonin receptor (5-HT3) antagonist, is primarily used as an antiemetic medication to prevent chemotherapy-induced nausea and vomiting [64]. While granisetron has been extensively studied in the context of managing chemotherapy-related symptoms, its specific role in directly targeting CSCs has not been investigated yet. Some studies have suggested that certain 5-HT3 receptor antagonists, including granisetron, may possess anti-CSC properties. These studies indicate that 5-HT3 receptor antagonists can modulate signaling pathways of CSCs [65, 66]. Here, for the first time, we demonstrated that a specific dosage of granisetron (300 μM) effectively inhibits BCSC properties in MCF7 cells and induces them to increase expression of the epithelial differentiation marker CD24, suggesting it acts as a differentiating agent in these cells.

A-443654 is a small molecule inhibitor that primarily targets AKT kinases, a protein family involved in multiple cellular signaling pathways regulating cell survival, proliferation and growth, the dysregulation of which has been implicated in various types of cancer [67]. Importantly, A-443654 has been shown to inhibit glioblastoma stem-like cells with similar efficacy compared with traditionally cultured glioblastoma cell lines [68], but there was still no research on its effects on BCSCs. In our study, we showed that it is effective in targeting MDA-MB-231-derived BCSCs with a weak differentiating effect.

Overall, the current study has important implications for the development of targeted therapies against BCSCs. The AI-driven identification of potential CSC differentiating agents expands the repertoire of molecules available for therapeutic interventions. The whole process is completely agnostic, eliminating the requirement for previous knowledge of specific molecular mechanisms to be targeted. Moreover, it highlights the power of AI in accelerating drug discovery and repurposing efforts, specifically in identifying molecules capable of targeting CSCs. By inducing CSC differentiation, these molecules hold the promise of reducing tumor heterogeneity, inhibiting self-renewal and sensitizing CSCs to conventional therapies. Nonetheless, in order to understand the potential impact on clinical applications of our study, some important limitations must be taken into account: (i) *in vitro* studies may not fully represent the complex and dynamic conditions of a living organism; (ii) cell lines might not accurately recapitulate the complexity of the original tumor; and (iii) *in vitro* studies often have short experimental durations, which may not capture the long-term effects of the drug or the development of drug resistance in CSCs over time. Concerning technical limitations, deep learning algorithms often pose significant computational challenges. DREDDA exhibited longer run times compared to alternative methods when the training process was performed from scratch (Table S7). Nonetheless, DREDDA’s pre-training phase is notably quicker than PA + cosine similarity, which necessitates dataset pre-transformation. Overall, given that both training and inference with DREDDA for the present application could be finished within minutes on a standard workstation, the involved computational burden should not hinder its practical use also on larger datasets. Finally, although the presented methodology is conceived to be applied to any biological context in which a target signature can be learned from single-cell data, its actual performance needs to be assessed in each specific application.

Future perspectives of this work involve the translation of these findings into preclinical and clinical studies. *In vivo* models and patient-derived xenograft models should be employed to assess the therapeutic efficacy, safety and pharmacokinetics of these compounds. Most of these molecules have already been tested on humans and considered safe, which constitutes an obvious advantage in terms of possible clinical translation. Additionally, further investigations are needed to elucidate the underlying molecular mechanisms by which these compounds induce CSC differentiation.

In conclusion, the integration of AI-driven screening and experimental validation provides a valuable approach to identifying molecules capable of differentiating BCSCs. The findings of this study, including the efficacy of triptolide, OTS-167, quinacrine, granisetron and A-443654, offer potential avenues for targeted therapies against BCSCs. This work lays the foundation for further research and development, bringing us closer to more effective and personalized treatments for BC patients.

## METHODS

### Gene expression data

A single-cell RNA-seq dataset consisting of 18 787 WTC-CRISPRi [37] hiPSCs was obtained from a previous study [37], in which each cell was assigned one of four pluripotency stages (core pluripotent, proliferative, early primed for differentiation, late primed for differentiation). The gene expression counts appeared both sparsely and skewly distributed, which may interfere with the artificial neural network model’s convergence. Therefore, a zero-inflated negative binomial (ZINB) autoencoder model [69] was used for normalization and denoising. Since it is an unsupervised method, the ZINB-based model was trained using both the source and the target datasets and the estimated mean parameter ($\underset{\_}{M}$) of the model was used as the denoised version of the expression count matrix. The denoised count matrix was then transformed with the mapping $x\to \mathit{\log}\left(x+\epsilon \right)$, where $\epsilon$ was set to $1.0\times{10}^{-5}$ to avoid undefined output values. Feature selection was performed on the source dataset by calculating the mutual information (MI) between each feature (gene) and the cluster labels. The top 1000 genes with the highest MI values were selected for subsequent analyses.

Concerning LINCS drug-induced profiles, the last release was obtained from GEO (ID: GSE70138). It includes 118 050 profiles obtained after treatment of 41 cell lines with 1796 small-molecule compounds. In this study, the level-5 data of the LINCS database were downloaded from the GEO website (GSE70138). CMAPPy2 (version 4.0.1) was used to access the GCTX data format. In particular, population-control normalized differential profiles included in the level-5 distribution were used. Finally, only genes included both in the LINCS profiles and in the set selected from the hiPSC data were used to train the computational model.

### Neural network model

The DREDDA architecture is a three-module composite deep neural network consisting of (1) a domain-specific autoencoder (green part of Figure 2A in the main text); (2) the main task classifier (blue part in Figure 2A); and (3) an adversarial domain classifier (red part in Figure 2A). The domain-specific autoencoder is an autoencoder with two independent encoders, one for each input dataset, and a shared decoder. The output of the decoder is sent to subsequent modules. The task classifier is a multi-layer perceptron providing classification probability for each of the four pluripotency stages. Its training is performed only on the source domain (hiPSC data) based on a cross-entropy loss function ${L}_{cls}$. The adversarial domain classifier receives the same input as the main classifier. However, it aims to provide a binary decision on whether such input comes from the source domain (hiPSCs) or the target domain (LINCS). Therefore, it is trained using both the source and target domain data with a binary cross-entropy loss function (${L}_{adv}$). Inspired by the Deep Domain Confusion framework [36], a third objective was introduced to enforce the similarity of intermediate network values between the source domain and the target domain examples by minimizing a Maximum Mean Discrepancy [70] (${L}_{dc}$). The whole model was trained to simultaneously optimize the three mentioned functions according to the composite loss function: ${L}_{cls}-{L}_{adv}+\mathrm{\lambda} {L}_{dc}$. The details of the network architecture and hyperparameters are listed in Table S8, shown in Figure S5 and described in Supplemental Methods.

Model training was performed with a two-phase update per training step: phase 1 updates the minimization objective parameters (parameters of the source domain encoder, the target domain encoder, the shared decoder and the task classifier), while phase 2 updates the maximization objective parameters (the adversarial domain classifier). For each training step, an equal number of source domain and target domain examples were sampled. The model was implemented using PyTorch 1.8 [71] deep learning framework and requires an NVIDIA CUDA-capable GPU with ≥10GB of memory.

### Performance evaluation

We evaluated the performance of DREDDA and three other alternative approaches through two commonly-used IR metrics: (1) the MRR and 2) the nDCG at four different thresholds (@50, 100, 150, 200). For a given ordered list of drugs $(l)$, e.g. the LINCS drugs ordered by the algorithm, and a target set $(s)$ of interest, e.g. the drugs with lowest DECCODE scores, the MRR is defined as:


$$ \mathrm{MRR}=\frac{1}{\mid \mathrm{s}\mid}\sum \limits_{i=1}^{\mid s\mid}\frac{1}{\operatorname{rank}\left[l,s\left[i\right]\right]} $$

where $\operatorname{rank}\left[l,s\left[i\right]\right]$ is the ranking of the ith drug of the target set $s$ in the ordered list $l$. The MRR metric is higher when a particular method prioritizes the drugs in the target set toward the beginning of the ordered list. The discounted cumulative gain ($\mathrm{DCG}@K$) metric is defined as:


$$ \mathrm{DCG}@K=\sum \limits_{i=1}^K\frac{\parallel \left[l\ \left[i\right]\ \mathrm{in}\ s\right]}{\log 2\ \left(i+1\right)} $$

where $K$ is a certain predefined threshold. The ideal discounted cumulative gain $\left(\mathrm{iDCG}@K\right)$ is defined as:


$$ \mathrm{DCG}@K=\sum \limits_{i=1}^K\frac{\parallel \left[\overline{l}\ \left[i\right]\ \mathrm{in}\ s\right]}{\log 2\ \left(i+1\right)} $$

where $\overline{l}$ is the reordered version of list $l$when all items in set $s$ are located in the first positions. Finally, the normalized discounted cumulative gain $\left(\mathrm{nDCG}@K\right)$ is defined as the ratio $\mathrm{DCG}@K/\mathrm{iDCG}@K$.

Alongside DREDDA, we evaluated the following methods as comparisons:

- *Random prediction*. LINCS profiles were randomly assigned a score that is uniformly distributed. The drug prioritizations is directly performed by ranking the random scores.
- *GEP + cosine similarity*. This strategy classifies the LINCS GEPs according to their average cosine similarity to the gene expression signature of the four hiPSC types.
- *PA + cosine similarity*. Following the same strategy as Golriz *et al*. [39], the LINCS profiles were converted to PAs using the Single-Sample Gene Set Enrichment Analysis [72, 73] and the cosine similarity was used on top of it for prediction.
- *GEP + Jaccard similarity*. Following a similar strategy as Engler *et al*. [74], the average similarity of LINCS GEPs to the gene expression signatures of the four hiPSC types was evaluated by the Jaccard similarity of the top 50 differentially expressed genes.
- *DREDDA w/o DA*. An ablated version of DREDDA, where domain adaptation mechanisms (including both adversarial training and domain confusion) were disabled.

Finally, we evaluated the diversity of each method. In our case, the prediction diversity of a particular method is defined as the entropy of the predicted label frequencies on the LINCS dataset normalized by the maximum entropy of a four-class categorial distribution.

**Key Points**

- AI predicts the ability of drugs to induce differentiation of CSCs using transcriptomics data.
- Domain adaptation allowed training on untreated cells and performing predictions on treated cells.
- Five molecules induced inhibition of CSCs in two BC cell lines, showing promising therapeutic potential.

## Supplementary Material

Supplementary_Material_bbae207

## Data Availability

All data needed to evaluate the conclusions in the paper are present in the paper and/or the Supplemental Materials. The code for the DREDDA model and relevant datasets for the reproduction of the results are available at https://github.com/lzx325/DREDDA.

## Author Biographies

**Zhongxiao Li** is a PhD student under the supervision of Professor Xin Gao at Structural and Functional Bioinformatics Group (SFB) at King Abdullah University of Science and Technology (KAUST).

**Antonella Napolitano** is a Research Fellow under the supervision of Monica Fedele at the Istituto di Endocrinologia e Oncologia Sperimentale (IEOS) of the National Council of the Research (CNR) in Italy.

**Monica fedele** is a group leader at the Istituto di Endocrinologia e Oncologia Sperimentale (IEOS) of the National Council of the Research (CNR) in Italy. Her main interest is in the role of chromatinic proteins in cancer.

**Xin Gao** is a Professor of Computer Science at the King Abdullah University of Science and Technology (KAUST), Saudi Arabia. His research interests include bioinformatics, computational biology, artificial intelligence and machine learning.

**Francesco Napolitano** is an Assistant Professor of Bioinformatics at the University of Sannio, Italy. His main research interest is in the use of artificial intelligence applied to omics data for drug discovery.
