# Supplementary material for: AI identifies potent inducers of breast cancer stem cell differentiation based on adversarial learning from gene expression data
Source: Brief Bioinform. 2024 May 2;25(3):bbae207. doi: 10.1093/bib/bbae207 (PMC11066897; doi:10.1093/bib/bbae207)
Supplement: Supplementary_Material_bbae207 [file supplementary_material_bbae207.pdf]

Supplemental Materials for "AI IDENTIFIES POTENT INDUCERS OF BREAST CANCER STEM CELL DIFFERENTIATION BASED ON ADVERSARIAL LEARNING FROM GENE EXPRESSION DATA"

Zhongxiao Li[1,2], Antonella Napolitano[3], Monica Fedele[3,*], Xin Gao[1,2,*], Francesco Napolitano[2,4,*]

1. Computer Science Program, Computer, Electrical and Mathematical Sciences and Engineering Division, King Abdullah University of Science and Technology (KAUST), Thuwal, Saudi Arabia.

2. Computational Bioscience Research Center, King Abdullah University of Science and Technology, Thuwal, Saudi Arabia.

3. Institute of Experimental Endocrinology and Oncology "G. Salvatore" (IEOS), CNR, 80131 Naples, Italy.

4. Dpt. of Science and Technology, University of Sannio, 82100 Benevento, Italy.

*corresponding authors: mfedele@unina.it (MF), xin.gao@kaust.edu.sa (XG), francesco.napolitano@unisannio.it (FN).

SUPPLEMENTAL METHODS

Detailed description of the DREDDA architecture

On the high level, the DREDDA architecture is a three-module composite deep neural network (DNN) model consisting of 1) A domain-specific autoencoder; 2) A task classifier; 3) An adversarial domain classifier (see **Figure 2** in the main text).

The domain-specific autoencoder is an autoencoder with two independent encoders and a shared decoder. Each of the two encoders processes profiles either from the source domain or the target domain. Here we denote them as $F_{enc\_s}(\cdot\,;\theta_{enc\_s})$ and $F_{enc\_t}(\cdot\,;\theta_{enc\_t})$, with θ_{enc_s} and θ_{enc_t} being their parameters, respectively. The use of two independent encoders yielded better performance as compared to a shared encoder (data not shown), likely due to the significant difference between the two input domains. With X_s denoting a source domain input and X_t denoting a target domain input, the two encoders then send the corresponding latent vectors $Z_s = F_{enc\_s}(X_s;\theta_{enc\_s})$ and $Z_t = F_{enc\_t}(X_t;\theta_{enc\_t})$ to the shared decoder, $F_{dec}(\cdot\,;\theta_{dec})$, for reconstruction. The reconstructed vectors X_s' and X_t' are used as inputs for the main task classifier and the adversarial task classifier.

The main task classifier is a multi-layer perceptron, here denoted as $F_{cls}(\cdot\,;\theta_{cls})$, estimating a classification probability for each of the four differentiation classes defined in the source domain, i.e. core, proliferative, early-primed, and late-primed. For convenience, here we use $F_{cls,L}(X';\theta_{cls})$ to represent the intermediate network representation after the L-th layer of the task classifier when X' is used as the input of the network.

The adversarial domain classifier, here denoted as $F_{adv}(\cdot\,;\theta_{adv})$ computes a binary decision on whether the reconstructed input comes from the source domain or the target domain.

Putting the three modules together, the DREDDA architecture aims to solve the so-called *unsupervised domain adaptation task* (1). Indeed, while ground truth labels (i.e., the cell cluster labels) are only available for the source domain to train the model, the actual aim is to apply the trained model to the target domain (i.e., the LINCS dataset). Compared with Ganin et al. (1), the DREDDA model includes several modifications to make it suitable for gene expression data.

In further detail, to train DREDDA on the unsupervised domain adaptation task, three objective (loss) functions were used. The first objective is the *task classification loss* for the classification task, which is a cross-entropy between prediction ($\hat{Y}$) ground truth label (Y, one-hot encoded):

$$L_{cls}^{(i)} = -\sum_{c}^{\#classes} Y_c^{(i)} \, log \, \hat{Y}_c^{(i)} \tag{1}$$

where $\hat{Y}^{(i)}$ is the output vector of the task classifier $F_{cls}\left(X^{(i)'}; \theta_{cls}\right)$ and $\hat{Y}_c^{(i)}$ takes its c-th dimension. The second objective is the *adversarial domain loss* for the adversarial domain classifier,

$$L_{adv}^{(i)} = -H^{(i)} \log \hat{H}^{(i)} \; -\left(1 - H^{(i)}\right) \log(1 - \hat{H}^{(i)}) \tag{2}$$

where $H^{(i)} = 0$ if the i-th example belongs to the source domain and $H^{(i)} = 1$ if it belongs to the target domain. $\hat{H}^{(i)}$ is the output of the adversarial domain classifier $F_{adv}(X^{(i)'}; \theta_{adv})$. Inspired by the Deep Domain Confusion (DDC) framework (2), we further introduced a third objective, the *domain confusion loss*, which explicitly enforces the similarity of intermediate network values between source domain and target domain examples by minimizing a Maximum Mean Discrepancy (MMD) (3) term,

$$\begin{aligned} MMD(\{source\}, \{target\}) \\ = \| \frac{1}{\#source} \sum_{i \in \{source\}} \phi(X_i) \\ - \frac{1}{\#target} \sum_{j \in \{target\}} \phi(X_j) \, \| \end{aligned} \tag{3}$$

where $\phi(X_i)$ is the intermediate value in the network after it takes as input X_i. We chose $\phi(X_i)$ to represent the L-th intermediate layer of the task classifier, i.e., $\phi(X_i) = F_{cls,L}(X_i{}'; \theta_{cls})$. Only the examples in one mini-batch are used to estimate MMD. The square of this term is used in the final objective function,

$$\begin{aligned} L = L_{cls} - L_{adv} + \lambda L_{dc} \\ = \left(\sum_{i \in \{source\}} L_{cls}^{(i)} \right) - \left(\sum_{i \in \{source\} \cup \{target\}} L_{adv}^{(i)} \right) \\ + \lambda MMD(\{source\}, \{target\})^2 \end{aligned} \tag{4}$$

The sign in front of L_{adv} is negative to maximize the domain adversarial classifier loss and thus discourage the use of domain-specific information during the training phase. The dependency of each loss term w.r.t. the model parameters can be inferred from the DREDDA architecture. Specifically, they are $L_{cls} = L_{cls}(\theta_{enc\_s}, \theta_{dec}, \theta_{cls})$, $L_{adv} = L_{adv}(\theta_{enc\_s}, \theta_{enc\_t}, \theta_{dec}, \theta_{adv})$ and $L_{dc} = L_{dc}(\theta_{enc\_s}, \theta_{enc\_t}, \theta_{dec}, \theta_{cls})$. The parameters are optimized via the following minimax objective,

$$L_{cls}(\theta_{enc\_s}, \theta_{dec}, \theta_{cls}) - L_{adv}(\theta_{enc\_s}, \theta_{enc\_t}, \theta_{dec}, \theta_{adv}) + L_{dc}(\theta_{enc\_s}, \theta_{enc\_t}, \theta_{dec}, \theta_{cls}) \quad (5)$$

Model Training and Implementation

Training of the network using the objective function described in Equation (5) can be done with a two-part update per training step. Specifically, one step updates the parameters that minimize the objective ($\theta_{enc\_s}, \theta_{enc\_t}, \theta_{dec}, \theta_{cls}$), while the other one updates the parameters that maximize the objective (θ_{adv}). For each training step, an equal number of source domain and target domain examples are sampled from the source domain and target domain datasets. Using the sampled examples, L_{cls} is computed using the sampled source domain examples, while L_{adv} and L_{dc} are computed using the sampled source and target domain examples. If plain minibatch stochastic gradient optimization is used, the parameters are updated as follows,

$$\theta_{enc\_s} \leftarrow \theta_{enc\_s} - \nabla_{\theta_{enc\_s}} L \theta_{enc\_t} \leftarrow \theta_{enc\_t} - \nabla_{\theta_{enc\_t}} L \quad (6)$$

$$\theta_{dec} \leftarrow \theta_{dec} - \nabla_{\theta_{dec}} L$$

$$\theta_{cls} \leftarrow \theta_{cls} - \nabla_{\theta_{cls}} L$$

$$\theta_{adv} \leftarrow \theta_{adv} + \nabla_{\theta_{adv}} L$$

Note that gradient *ascend* is performed on θ_{adv} while gradient *descent* is performed on all other parameters. The model is implemented using PyTorch 1.8 (4) deep learning framework and can be run on any NVIDIA CUDA-capable GPUs with $\geq 10GB$ memory.

Model Testing and Predictions

After each training epoch, the model's performance was evaluated in the source domain based on multi-class classification loss and accuracy. At the same time, the adversarial classifier was evaluated on the full dataset based on the ability to discriminate between data points coming from the source domain and the target domain. The model was optimized towards the highest source domain accuracy, with target domain accuracy within the 40%-60% range. The trained model was then applied to score each profile in the LINCS database and a final unique drug-related score was obtained by averaging over all the scores obtained for the same drug across different cell lines.

Gene Set Enrichment Analysis of DREDDA-prioritized drugs

Drug-Set Enrichment Analysis (DSEA) (5), which is built on the classical Gene Set Enrichment Analysis (GSEA) (6), identifies pathways consistently dysregulated across a set of experimental conditions (such as drug treatments). DSEA was performed using the top 30 drugs prioritized by DREDDA as the foreground set and the bottom 30 as the background set on the LINCS level 5 dataset for the “C5 GO Biological Process” and “C5 GO Molecular Function” categories as obtained from the MsigDB v7.2 (7). The analysis was performed using the gep2pep R package (8). Summaries for the positive and negative expression within families of pathways were obtained by counting the pathways with positive or negative enrichment scores that were found significant ($p < 0.05$) by the DSEA analysis and fell below a given level of the GO category. In this case, negative enrichments for GO terms starting with “Negative” (such as “Negative regulation of cell differentiation”) were counted as positive.

Cell cultures and treatments

Human breast cancer MCF7 and MDA-MB-231 cell lines (American Type Culture Collection, ATCC, Manassas, VA) were grown in Dulbecco’s modified Eagle’s medium (DMEM, Sigma-Aldrich, Milan, Italy) and Roswell Park Memorial Institute-1640 medium (RPMI-1640, Sigma-Aldrich), respectively, supplemented with 10% heat-inactivated fetal bovine serum (FBS, Sigma-Aldrich) and 1%penicillin–streptomycin, in 95% air/5% CO2 atmosphere at 37 °C.

The drugs, IC50, and working concentrations for cell treatments are shown in **Table S5**. All drugs were dissolved in DMSO (stock solution) and further diluted in a cell medium (working solution). The negative control was the solvent alone at its final concentration.

Cell viability

Cell viability was assessed by CellTiter 96 AQueous One Solution Cell Proliferation Assay (Promega BioSciences Inc., San Luis Obispo, CA), according to the manufacturer’s instructions, 72 hours after cell treatment (5×10^3 cells/well, 96-well plates). Each drug concentration was tested in at least three independent experiments for each molecule and each cell line.

Mammosphere assay and CSC self-renewal

Cells were seeded at sub-confluence in 6-well plates. After 16 hrs they were treated with two different concentrations of each compound or DMSO (drug solvent) as negative control. At 24 hrs post-treatment, cells were washed out of the serum and seeded (3000 cells/well) in 24-well low attachment plates with stem cell medium, consisting of 1% B27 (Invitrogen, Carlsbad, CA), 10ng/ml bFGF (Invitrogen), 20 ng/ml EGF (Sigma-Aldrich) in DMEM/F12 (SIGMA-Aldrich) supplemented with 1% penicillin/streptomycin) to form primary (P0) mammospheres. The number and diameter of P0 mammospheres were counted after a period of 7-14 days to estimate the number of treatment-resistant CSCs. Moreover, their diameter was measured to analyze their capacity to proliferate. Then, spheres were disaggregated, and single cells were replated (3000 cells/well) to form

secondary (P1) mammospheres. P1 mammospheres were analyzed after further 7-14 days to calculate the CSC self-renewal (P1 mammospheres/P0 plated single cells x 100).

FACS analysis

Adherent cells were treated for 24 hrs with two different concentrations of each compound or negative control (DMSO), then cells were washed with phosphate-buffered saline (PBS) and harvested with 0.05% trypsin/0.025% EDTA. Detached cells were washed and resuspended in PBS supplemented with 2% FBS, 0.2% sodium azide (Staining buffer). Combinations of fluorochrome-conjugated monoclonal antibodies against human CD44 (PE; Santa Cruz cat. # SC-18849-PE) and CD24 (FITC; Beckton Dickinson cat. # BD555427) were added to the cell suspension (1 x 10^6 cells) and incubated at 4°C in the dark for 30 min. The labeled cells were washed in a staining buffer and then analyzed on a BD Accuri Flow Cytometer (BD).

Statistics

Ordinary one-way analysis of variance (ANOVA) followed by Tukey's multiple comparisons test, through GraphPad Prism 6 software, was applied for the comparison of groups of experimental data. Values analyzed are the average +/- SD of at least three independent experiments.

SUPPLEMENTAL FIGURES

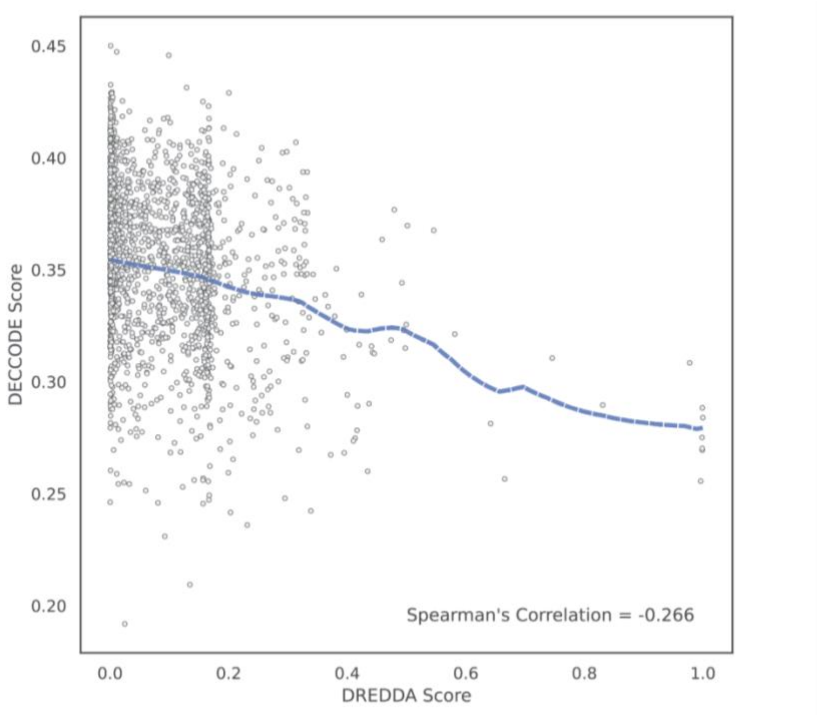

Figure S1 Comparison between DREDDA scores (predicting differentiation) and DECCODE scores (predicting stemness). Although at low DREDDA scores, there is no clear correlation between the two measures, high DREDDA scores tend to correspond to low DECCODE scores and high DECCODE scores tend to correspond to low DREDDA scores.

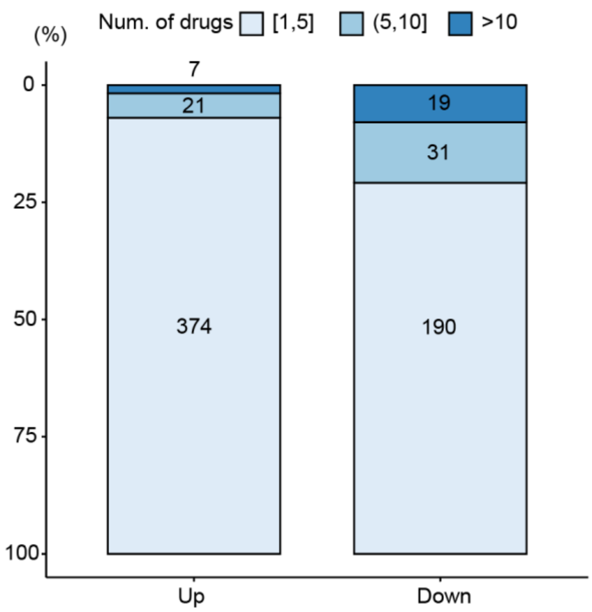

Figure S2 The number and proportion of genes that are commonly up- (down-) regulated by the top-30 drugs.

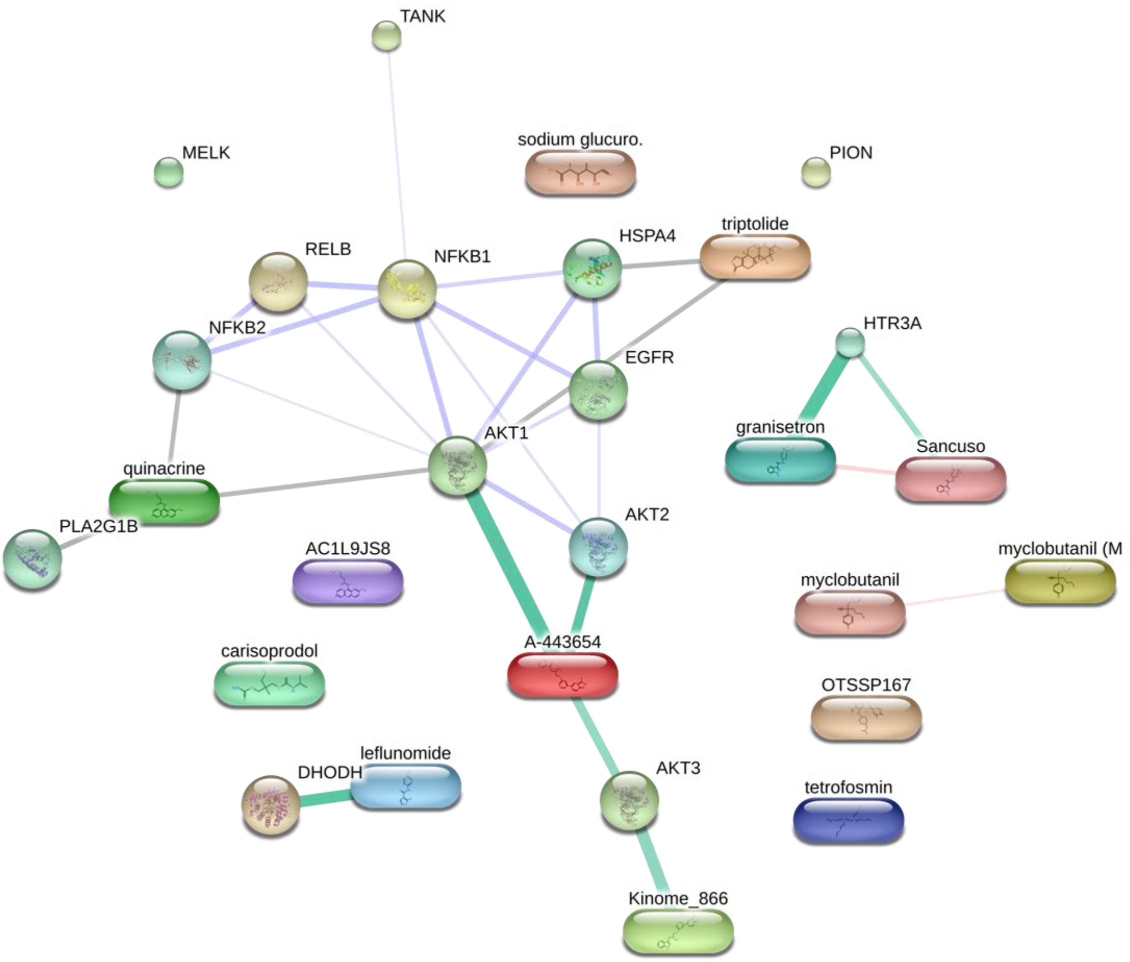

Figure S3 Interaction network between the tested drugs and their known targets (analysis performed using the STITCH tool (9))

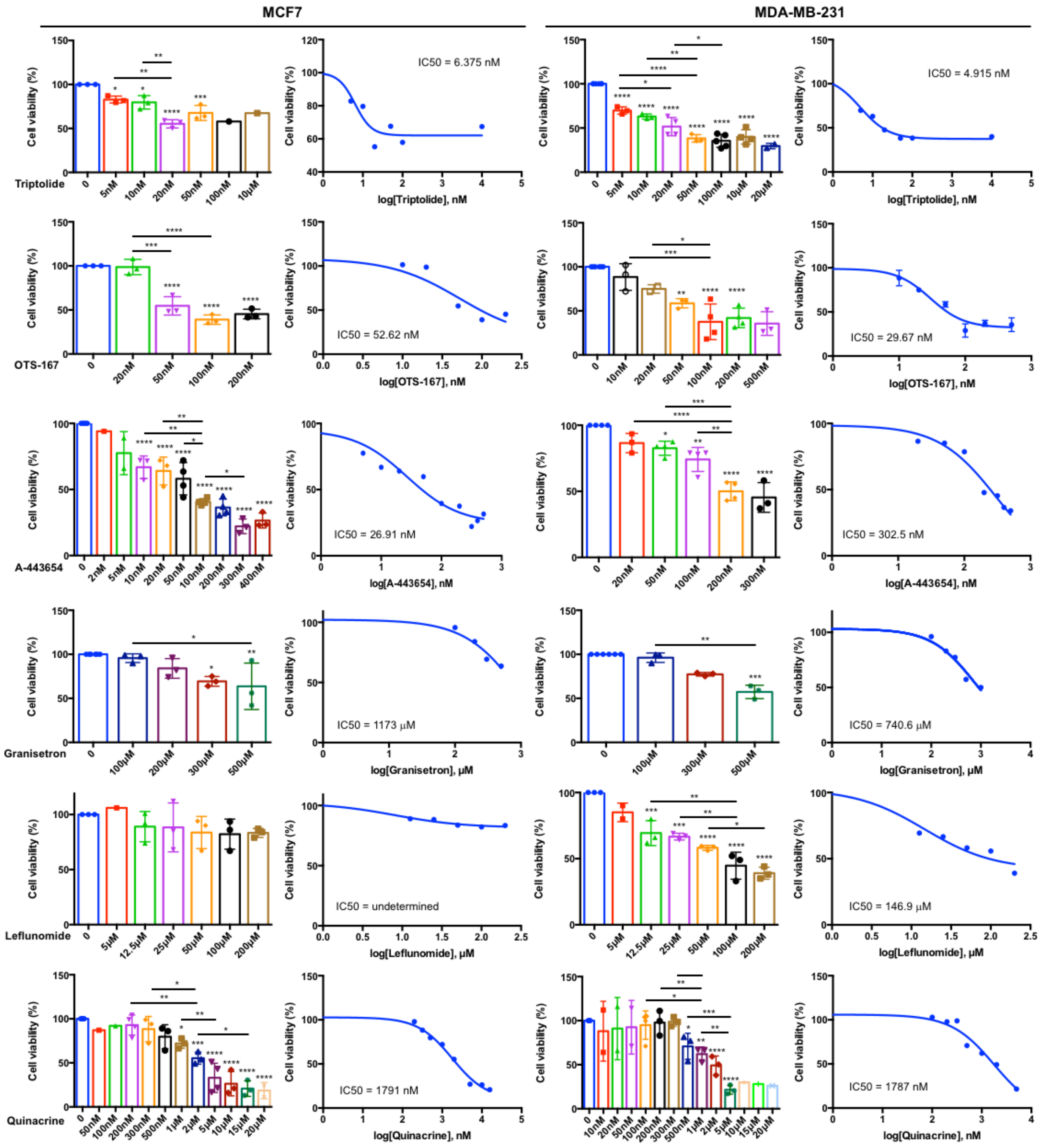

Figure S4 Percentage of cell viability and IC50 of MCF7 and MDA-MB-231 cells after exposure to increasing drug concentrations. MTT test results are reported as a percentage of cell viability. Each value was normalized with respect to its control. Dose-response curves were used to generate IC50. Statistics and IC50 curves were performed using Prism GraphPad 6.0. *, $P < 0.05$; **, $P < 0.01$; ***, $P < 0.001$; ****, $P < 0.0001$

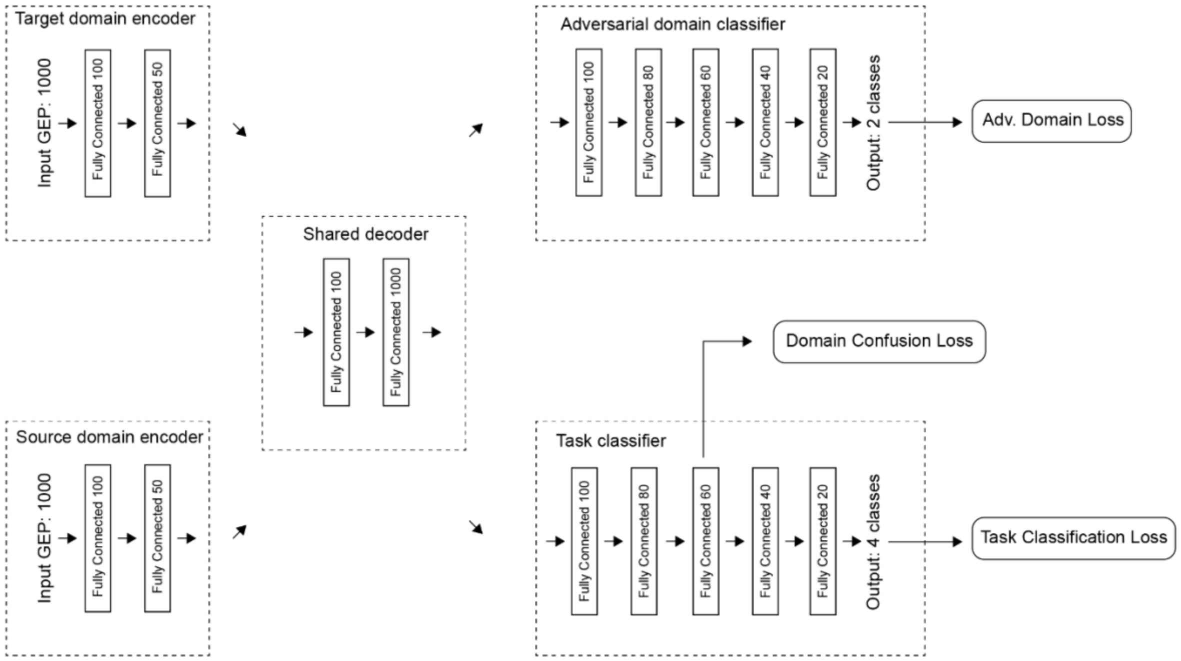

Figure S5 Detailed schema of the DREDDA architecture

SUPPLEMENTAL TABLES

Table S1 Top 30 molecules as prioritized by DREDDA.

#	Drug	2D structure	Functional Category	Notes
1	triptolide	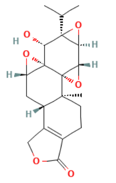	Anti-proliferative agent	Has a role as an antispermatogenic agent and a plant metabolite. - ChEBI - http://www.ebi.ac.uk/chebi/searchId.do?chebiId=CHEBI:9747
2	OTS-167	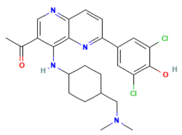	MELK inhibitor	A naphthyridine derivative. - ChEBI - http://www.ebi.ac.uk/chebi/searchId.do?chebiId=CHEBI:95088
3	CGP-60474	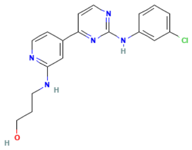	CDK inhibitor	A substituted aniline. - ChEBI - http://www.ebi.ac.uk/chebi/searchId.do?chebiId=CHEBI:91339
4	dinaciclib	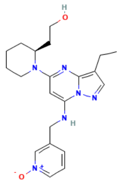	CDK inhibitor	A pyrazolopyrimidine - ChEBI - http://www.ebi.ac.uk/chebi/searchId.do?chebiId=CHEBI:95060
5	WZ-3105	-	Small molecule kinase inhibitors	-
6	alvocidib	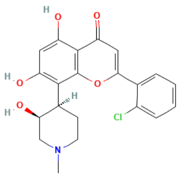	CDK9 kinase inhibitor	A cyclin-dependent kinase 9 (CDK9) inhibitor, it has been studied for the treatment of acute myeloid leukaemia, arthritis and atherosclerotic plaque formation. It has a role as an antineoplastic agent, an EC 2.7.11.22 (cyclin-dependent kinase) inhibitor, an antirheumatic drug and an apoptosis inducer. - ChEBI - http://www.ebi.ac.uk/chebi/searchId.do?chebiId=CHEBI:47344
7	BMS-387032	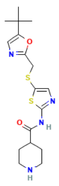	CDK2 inhibitor	An ATP-competitive inhibitor of CDK2, CDK7 and CDK9 kinases and exhibits anti-cancer properties. It has a role as an apoptosis inducer, an antineoplastic agent, an EC 2.7.11.22 (cyclin-dependent kinase) inhibitor and an angiogenesis inhibitor. - ChEBI - http://www.ebi.ac.uk/chebi/searchId.do?chebiId=CHEBI:91399

#	Drug	2D structure	Functional Category	Notes
8	staurosporine	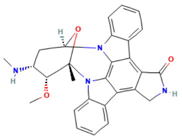	General protein kinase inhibitor	Has a role as an EC 2.7.11.13 (protein kinase C) inhibitor, a geroprotector, a bacterial metabolite and an apoptosis inducer.<br>- ChEBI -<br>http://www.ebi.ac.uk/chebi/searchId.do?chebiId=CHEBI:15738
9	AT-7519	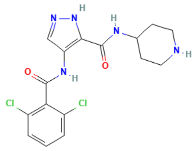	CDK inhibitor	Has a role as an EC 2.7.11.22 (cyclin-dependent kinase) inhibitor and an antineoplastic agent.<br>- ChEBI -<br>http://www.ebi.ac.uk/chebi/searchId.do?chebiId=CHEBI:91326
10	A-443654	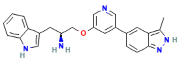	Pan-Akt inhibitor	A member of indoles<br>- ChEBI -<br>http://www.ebi.ac.uk/chebi/searchId.do?chebiId=CHEBI:91351
11	JNK-9L	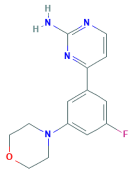	JNK inhibitor	
12	mitoxantrone	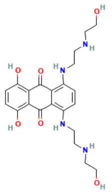	TOPO II inhibitor	Has a role as an antineoplastic agent and an analgesic.<br>- ChEBI -<br>http://www.ebi.ac.uk/chebi/searchId.do?chebiId=CHEBI:50729
13	Ro-4987655	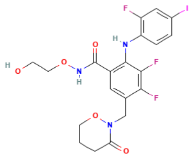	MEK inhibitor	
14	binimetinib	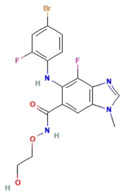	MEK inhibitor	A MEK1 and MEK2 inhibitor (IC50= 12 nM). Approved by the FDA for the treatment of patients with unresectable or metastatic melanoma with a BRAF V600E or V600K mutation in combination with encorafenib. It has a role as an EC 2.7.11.24 (mitogen-activated protein kinase) inhibitor, an antineoplastic agent and an apoptosis inducer.<br>- ChEBI -<br>http://www.ebi.ac.uk/chebi/searchId.do?chebiId=CHEBI:145371

#	Drug	2D structure	Functional Category	Notes
15	R-547	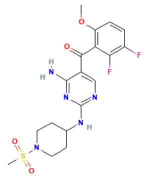	CDK inhibitor	
16	bardoxolone-methyl	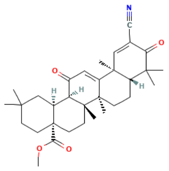	NF-κB pathway inhibitor	Bardoxolone methyl is a member of cyclohexenones.<br>- ChEBI -<br>http://www.ebi.ac.uk/chebi/searchId.do?chebiId=CHEBI:177406
17	PF-431396	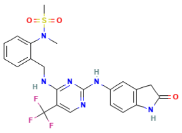	FAK and PYK2 inhibitor	A sulfonamide.<br>- ChEBI<br><br>http://www.ebi.ac.uk/chebi/searchId.do?chebiId=CHEBI:91388
18	camicinal	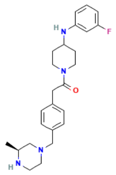	Motilin agonist	Camicinal is a member of acetamides.<br>- ChEBI -<br>http://www.ebi.ac.uk/chebi/searchId.do?chebiId=CHEBI:177624
19	AZD-5438	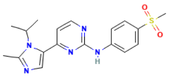	CDK inhibitor	A sulfonamide.<br>- ChEBI -<br>http://www.ebi.ac.uk/chebi/searchId.do?chebiId=CHEBI:91419
20	epirubicin	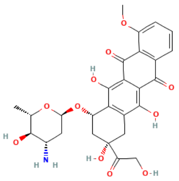	DNA intercalating agents	Has a role as an EC 5.99.1.3 [DNA topoisomerase (ATP-hydrolysing)] inhibitor, an antineoplastic agent and an antimicrobial agent.<br>- ChEBI -<br>http://www.ebi.ac.uk/chebi/searchId.do?chebiId=CHEBI:47898
21	AS-601245	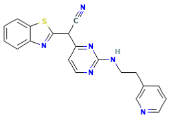	JNK inhibitor	A member of benzothiazoles<br>- ChEBI -<br>http://www.ebi.ac.uk/chebi/searchId.do?chebiId=CHEBI:91345
22	AZD-8330	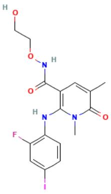	MEK inhibitor	A pyridinecarboxamide. It is functionally related to a nicotinamide.<br>- ChEBI -<br>http://www.ebi.ac.uk/chebi/searchId.do?chebiId=CHEBI:91424

#	Drug	2D structure	Functional Category	Notes
23	leflunomide	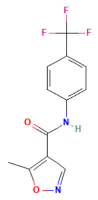	Pyrimidine synthesis inhibitor	Has a role as a non-steroidal anti-inflammatory drug, an antineoplastic agent, an antiparasitic agent, an EC 1.3.98.1 [dihydroorotate oxidase (fumarate)] inhibitor, a hepatotoxic agent, a prodrug, a pyrimidine synthesis inhibitor, an immunosuppressive agent, an EC 3.1.3.16 (phosphoprotein phosphatase) inhibitor and a tyrosine kinase inhibitor. - ChEBI - http://www.ebi.ac.uk/chebi/searchId.do?chebiId=CHEBI:6402
24	bruceantin	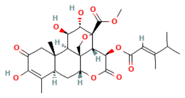	Protein and DNA synthesis inhibitor	Bruceantin is a triterpenoid. - ChEBI - http://www.ebi.ac.uk/chebi/searchId.do?chebiId=CHEBI:3188
25	SNX-2112	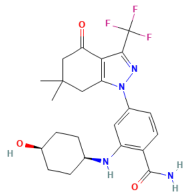	Anti-proliferative agent	
26	PF-03758309	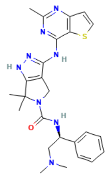	PAK4 Inhibitor	An organic heterobicyclic compound, an organosulfur heterocyclic compound and an organonitrogen heterocyclic compound. - ChEBI - http://www.ebi.ac.uk/chebi/searchId.do?chebiId=CHEBI:93751
27	rebastinib	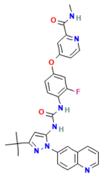	TIE2 kinase inhibitor	Has a role as a tyrosine kinase inhibitor. - ChEBI - http://www.ebi.ac.uk/chebi/searchId.do?chebiId=CHEBI:62166
28	granisetron	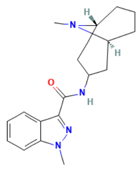	Serotonin 5-HT3 receptor antagonist	A selective 5-HT3 receptor antagonist, it is used (generally as the monohydrochloride salt) to manage nausea and vomiting caused by cancer chemotherapy and radiotherapy, and to prevent and treat postoperative nausea and vomiting. It has a role as a serotonergic antagonist and an antiemetic. - ChEBI - http://www.ebi.ac.uk/chebi/searchId.do?chebiId=CHEBI:5537
29	AEE-788	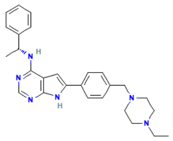	RTK inhibitor	A potent inhibitor of human EGFR, VEGFR and HER2 receptor tyrosine kinases and exhibits anticancer and antiangiogenic activity. It has a role as an epidermal growth factor receptor antagonist, an EC 2.7.10.1 (receptor protein-tyrosine kinase) inhibitor, an antineoplastic agent, an angiogenesis inhibitor, a trypanocidal drug and an apoptosis inducer.

#	Drug	2D structure	Functional Category	Notes
				- ChEBI - http://www.ebi.ac.uk/chebi/searchId.do?chebiId=CHEBI:40629
30	deoxycholic-acid	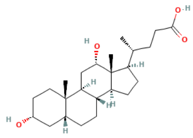	Oxidative agent	A bile acid, a dihydroxy-5beta-cholanic acid and a C24-steroid. It is a conjugate acid of a deoxycholate. - ChEBI - http://www.ebi.ac.uk/chebi/searchId.do?chebiId=CHEBI:28834

Table S2 Genes that are commonly up- or down- regulated by the top 30 molecules.

Gene	# Drugs up-regulates	# Drugs down-regulates	Up-regulating drug names	Down-regulating drug names
LYN	17	0	triptolide,CGP-60474,dinaciclib,OTS-167,BMS-387032,WZ-3105,alvocidib,JNK-9L,AT-7519,A-443654,AZD-5438,staurosporine,PF-431396,AS-601245,PF-562271,rebastinib,BMS-345541	
XIST	14	0	OTS-167,JNK-9L,AT-7519,R-547,A-443654,epirubicin,AZD-5438,staurosporine,PF-431396,AS-601245,PF-562271,rebastinib,BMS-345541,fadrozole	
COL11A1	14	0	CGP-60474,OTS-167,WZ-3105,alvocidib,JNK-9L,R-547,epirubicin,mitoxantrone,AZD-5438,PF-431396,rebastinib,BMS-345541,ivacaftor,fadrozole	
EGR1	13	2	CGP-60474,dinaciclib,OTS-167,BMS-387032,WZ-3105,JNK-9L,AT-7519,R-547,PF-431396,AS-601245,PF-562271,bardoxolone-methyl,BMS-345541	Ro-4987655,binimetinib
E2F2	12	0	CGP-60474,BMS-387032,JNK-9L,AT-7519,A-443654,PF-431396,AS-601245,PF-562271,PHA-767491,BMS-345541,ivacaftor,fadrozole	
IKZF1	12	0	CGP-60474,BMS-387032,WZ-3105,JNK-9L,AT-7519,A-443654,mitoxantrone,PF-431396,PF-562271,BMS-345541,ivacaftor,fadrozole	
GPC1	12	0	CGP-60474,dinaciclib,OTS-167,alvocidib,AT-7519,R-547,epirubicin,mitoxantrone,AZD-5438,PF-03758309,bardoxolone-methyl,PHA-767491	
DNAJB1	2	14	bardoxolone-methyl,MG-132	CGP-60474,dinaciclib,OTS-167,BMS-387032,WZ-3105,alvocidib,JNK-9L,AT-7519,A-443654,mitoxantrone,AZD-5438,staurosporine,PF-431396,AS-601245
PCNA	1	22	fadrozole	triptolide,CGP-60474,dinaciclib,OTS-167,BMS-387032,WZ-3105,alvocidib,JNK-9L,AT-7519,R-547,A-443654,staurosporine,PF-431396,AS-601245,PF-562271,PF-03758309,rebastinib,PHA-848125,PHA-767491,ivacaftor,BGT-226,MG-132
HSPA8	0	23		triptolide,CGP-60474,dinaciclib,OTS-167,BMS-387032,WZ-3105,alvocidib,JNK-9L,AT-7519,A-443654,epirubicin,mitoxantrone,AZD-5438,Ro-4987655,staurosporine,PF-431396,AS-601245,PF-562271,rebastinib,PHA-848125,PHA-767491,ivacaftor,binimetinib
CCNB2	0	23		triptolide,CGP-60474,dinaciclib,OTS-167,BMS-387032,alvocidib,JNK-9L,AT-7519,R-547,epirubicin,mitoxantrone,AZD-

				5438,staurosporine,PF-431396,AS-601245,PF-562271,PF-03758309,rebastinib,bardoxolone-methyl,PHA-848125,PHA-767491,BGT-226,MG-132
CDC25B	0	21		CGP-60474,dinaciclib,OTS-167,BMS-387032,WZ-3105,alvocidib,JNK-9L,AT-7519,R-547,A-443654,mitoxantrone,AZD-5438,staurosporine,PF-431396,AS-601245,PF-562271,rebastinib,PHA-848125,PHA-767491,BMS-345541,MG-132
TOP2A	0	20		triptolide,dinaciclib,OTS-167,BMS-387032,WZ-3105,alvocidib,AT-7519,R-547,epirubicin,mitoxantrone,AZD-5438,Ro-4987655,PF-431396,PF-03758309,rebastinib,bardoxolone-methyl,PHA-848125,binimetinib,BGT-226,MG-132
CDC20	0	20		triptolide,CGP-60474,dinaciclib,OTS-167,BMS-387032,alvocidib,JNK-9L,AT-7519,R-547,epirubicin,mitoxantrone,AZD-5438,Ro-4987655,staurosporine,PF-431396,PF-562271,PF-03758309,rebastinib,PHA-848125,binimetinib
TIMM9	0	18		triptolide,CGP-60474,dinaciclib,OTS-167,BMS-387032,WZ-3105,alvocidib,AT-7519,epirubicin,mitoxantrone,PF-431396,AS-601245,PF-562271,rebastinib,bardoxolone-methyl,BMS-345541,ivacaftor,MG-132
CCNA2	0	16		triptolide,WZ-3105,alvocidib,AT-7519,R-547,A-443654,epirubicin,mitoxantrone,AZD-5438,staurosporine,PF-431396,PF-562271,PF-03758309,PHA-848125,BGT-226,MG-132
CANT1	0	15		CGP-60474,dinaciclib,BMS-387032,WZ-3105,alvocidib,JNK-9L,AT-7519,A-443654,mitoxantrone,staurosporine,PF-431396,AS-601245,PF-562271,PHA-767491,BMS-345541
TXLNA	0	14		triptolide,CGP-60474,dinaciclib,OTS-167,BMS-387032,WZ-3105,alvocidib,JNK-9L,AT-7519,AZD-5438,staurosporine,AS-601245,rebastinib,PHA-767491
KIF20A	0	14		R-547,epirubicin,mitoxantrone,AZD-5438,Ro-4987655,staurosporine,PF-431396,PF-562271,PF-03758309,rebastinib,PHA-848125,fadrozole,binimetinib,MG-132
KEAP1	0	13		CGP-60474,BMS-387032,WZ-3105,alvocidib,JNK-9L,AT-7519,AZD-5438,staurosporine,PF-431396,PF-562271,rebastinib,PHA-767491,BMS-345541
TRAPPC3	0	13		triptolide,CGP-60474,dinaciclib,OTS-167,BMS-387032,WZ-3105,alvocidib,AT-7519,PF-431396,AS-601245,PF-562271,rebastinib,BMS-345541
SMC4	0	12		triptolide,AT-7519,R-547,epirubicin,AZD-5438,staurosporine,PF-431396,PF-562271,PF-03758309,rebastinib,PHA-848125,binimetinib
CRK	0	12		CGP-60474,BMS-387032,WZ-3105,alvocidib,JNK-9L,AT-7519,PF-

				431396,AS-601245,PF-562271,rebastinib,BMS-345541,ivacaftor
USP22	0	12		CGP-60474,dinaciclib,OTS-167,BMS-387032,WZ-3105,alvocidib,JNK-9L,AT-7519,A-443654,mitoxantrone,AS-601245,PHA-767491
C2CD5	0	11		triptolide,OTS-167,AT-7519,R-547,AZD-5438,PF-431396,PF-03758309,bardoxolone-methyl,PHA-767491,BMS-345541,MG-132
PUF60	0	11		CGP-60474,dinaciclib,OTS-167,BMS-387032,WZ-3105,alvocidib,JNK-9L,mitoxantrone,AS-601245,PF-562271,rebastinib

Table S3 Pathways enriched by genes commonly dysregulated by the top 10 drugs.

Category	Term	Count	%	P-Value	Benjamini
GOTERM_CC_DIRECT	nucleoplasm	16	61,5	8,8E-6	1,1E-3
GOTERM_BP_DIRECT	cell cycle	5	19,2	1,0E-3	2,6E-1
GOTERM_CC_DIRECT	cyclin-dependent protein kinase holoenzyme complex	3	11,5	1,2E-3	7,7E-2
GOTERM_BP_DIRECT	cell division	5	19,2	1,4E-3	2,6E-1
GOTERM_CC_DIRECT	cytosol	15	57,7	2,0E-3	8,4E-2
GOTERM_CC_DIRECT	ribonucleoprotein complex	4	15,4	4,8E-3	1,2E-1
GOTERM_CC_DIRECT	nucleus	15	57,7	4,8E-3	1,2E-1
GOTERM_MF_DIRECT	protein binding	23	88,5	5,8E-3	3,0E-1
GOTERM_CC_DIRECT	centrosome	5	19,2	7,5E-3	1,5E-1
GOTERM_MF_DIRECT	cadherin binding	4	15,4	7,5E-3	3,0E-1
GOTERM_MF_DIRECT	chaperone binding	3	11,5	7,8E-3	3,0E-1
GOTERM_MF_DIRECT	enzyme binding	4	15,4	1,1E-2	3,3E-1
GOTERM_BP_DIRECT	cellular response to insulin-like growth factor stimulus	2	7,7	1,2E-2	1,0E0
GOTERM_BP_DIRECT	regulation of cell adhesion mediated by integrin	2	7,7	1,7E-2	1,0E0
GOTERM_BP_DIRECT	cellular response to nitric oxide	2	7,7	1,9E-2	1,0E0
GOTERM_BP_DIRECT	estrous cycle	2	7,7	2,1E-2	1,0E0
GOTERM_MF_DIRECT	DNA binding	6	23,1	2,5E-2	5,0E-1
GOTERM_BP_DIRECT	mitotic cell cycle phase transition	2	7,7	2,8E-2	1,0E0

Category	Term	Count	%	P-Value	Benjamini
GOTERM_MF_DIRECT	histone acetyltransferase binding	2	7,7	3,0E-2	5,0E-1
GOTERM_MF_DIRECT	protein domain specific binding	3	11,5	3,1E-2	5,0E-1
GOTERM_MF_DIRECT	ephrin receptor binding	2	7,7	3,5E-2	5,0E-1
GOTERM_MF_DIRECT	cyclin-dependent protein serine/threonine kinase regulator activity	2	7,7	4,0E-2	5,1E-1
GOTERM_BP_DIRECT	chaperone mediated protein folding requiring cofactor	2	7,7	4,1E-2	1,0E0
GOTERM_BP_DIRECT	positive regulation of mitotic cell cycle	2	7,7	4,4E-2	1,0E0
GOTERM_CC_DIRECT	extracellular exosome	7	26,9	4,8E-2	8,5E-1
GOTERM_CC_DIRECT	cytoplasm	12	46,2	5,6E-2	8,6E-1
GOTERM_BP_DIRECT	regulation of DNA replication	2	7,7	5,8E-2	1,0E0
GOTERM_MF_DIRECT	ubiquitin protein ligase binding	3	11,5	5,9E-2	6,5E-1
GOTERM_BP_DIRECT	regulation of cyclin-dependent protein serine/threonine kinase activity	2	7,7	6,1E-2	1,0E0
GOTERM_MF_DIRECT	protein binding involved in protein folding	2	7,7	6,3E-2	6,5E-1
GOTERM_BP_DIRECT	regulation of signal transduction	2	7,7	6,3E-2	1,0E0
GOTERM_BP_DIRECT	ephrin receptor signaling pathway	2	7,7	6,3E-2	1,0E0
GOTERM_BP_DIRECT	G2/M transition of mitotic cell cycle	2	7,7	6,3E-2	1,0E0
GOTERM_CC_DIRECT	male germ cell nucleus	2	7,7	6,6E-2	9,0E-1
GOTERM_BP_DIRECT	cellular response to heat	2	7,7	6,9E-2	1,0E0
GOTERM_BP_DIRECT	erythrocyte differentiation	2	7,7	6,9E-2	1,0E0
GOTERM_BP_DIRECT	response to unfolded protein	2	7,7	7,1E-2	1,0E0
GOTERM_CC_DIRECT	chromosome, centromeric region	2	7,7	7,4E-2	9,1E-1
GOTERM_MF_DIRECT	receptor tyrosine kinase binding	2	7,7	8,1E-2	7,5E-1
GOTERM_MF_DIRECT	scaffold protein binding	2	7,7	8,5E-2	7,5E-1
GOTERM_BP_DIRECT	hematopoietic progenitor cell differentiation	2	7,7	9,2E-2	1,0E0
GOTERM_BP_DIRECT	response to insulin	2	7,7	9,3E-2	1,0E0

Category	Term	Count	%	P-Value	Benjamini
GOTERM_BP_DIRECT	rhythmic process	2	7,7	9,5E-2	1,0E0

Table S4 Top-enriched GO terms under the Biological Process category resulting from drug set enrichment analysis (DSEA) of the top 30 drugs vs bottom 30 drugs.

Pathway	ES	PVadj
Morphogenesis of a polarized epithelium	-0.97	1.11E-10
Nuclear envelope organization	-0.97	1.11E-10
Female meiosis I	-0.97	1.11E-10
Dosage compensation	-0.97	1.11E-10
Pyrimidine containing compound salvage	-0.97	1.11E-10
Protein import	-0.97	1.11E-10
RNA 3’ end processing	-0.97	1.11E-10
MRNA 3’ end processing	-0.97	1.11E-10
Positive regulation of type I interferon production	-0.97	1.11E-10
RNA stabilization	-0.97	1.11E-10
Cell cycle G2-M phase transition	-0.97	1.11E-10
tRNA transport	-0.97	1.11E-10
Nuclear transport	-0.97	1.11E-10
Telomerase RNA localization	-0.97	1.11E-10
ncRNA export from nucleus	-0.97	1.11E-10
Regulation of protein localization to nucleus	-0.97	1.11E-10
Regulation of signal transduction by p53 class mediator	-0.97	1.11E-10
Positive regulation of cyclin dependent protein kinase activity	-0.97	1.11E-10
Regulation of protein localization to chromosome telomeric region	-0.97	1.11E-10
Positive regulation of telomerase rna localization to cajal body	-0.97	1.11E-10

Photoreceptor cell maintenance	0.97	1.11E-10
Pattern specification process	0.93	3.00E-09
Hormone transport	0.93	3.00E-09
Monovalent inorganic cation transport	0.93	3.00E-09
Hormone metabolic process	0.93	3.00E-09
Activation of janus kinase activity	0.93	3.00E-09
Photoreceptor cell differentiation	0.93	3.00E-09
Regulation of calcium ion transmembrane transport	0.93	3.00E-09
Regulation of neurotransmitter levels	0.9	5.79E-08
Eye photoreceptor cell differentiation	0.9	5.79E-08
Regionalization	0.9	5.79E-08
Regulation of systemic arterial blood pressure	0.9	5.79E-08
Potassium ion transport	0.9	5.79E-08
Excretion	0.9	5.79E-08
Regulation of blood pressure	0.9	5.79E-08
Specification of symmetry	0.9	5.79E-08
Regulation of hormone levels	0.9	5.79E-08
Response to auditory stimulus	0.9	5.79E-08
Organic anion transport	0.9	5.79E-08
Organic hydroxy compound transport	0.9	5.79E-08

Table S5 Top-enriched GO terms under the Cellular Component category resulting from drug set enrichment analysis (DSEA) of the top 30 drugs vs bottom 30 drugs.

Pathway	ES	PVadj
Nuclear periphery	-1.00	0.00E+00
Nuclear ubiquitin ligase complex	-0.97	1.11E-12
Fibrillar center	-0.97	1.11E-12
Nuclear matrix	-0.97	1.11E-12
Aminoacyl trna synthetase multienzyme complex	-0.97	1.11E-12
Protein acetyltransferase complex	-0.97	1.11E-12
Nuclear membrane	-0.97	1.11E-12
Histone deacetylase complex	-0.93	3.00E-11
Commitment complex	-0.93	3.00E-11
Transcription export complex	-0.93	3.00E-11
Chromosome centromeric region	-0.93	3.00E-11
Kinetochore	-0.93	3.00E-11
Condensed chromosome centromeric region	-0.93	3.00E-11
Spindle pole	-0.93	3.00E-11
Nuclear envelope	-0.93	3.00E-11
Rna polymerase III complex	-0.93	3.00E-11
Spliceosomal complex	-0.93	3.00E-11
U1 snRNP	-0.93	3.00E-11
Centrosome	-0.93	3.00E-11
Spindle	-0.93	3.00E-11
Cilium	0.93	3.00E-11

Photoreceptor outer segment	0.90	5.79E-10
Intrinsic component of the cytoplasmic side of the plasma membrane	0.90	5.79E-10
Photoreceptor connecting cilium	0.90	5.79E-10
Interphotoreceptor matrix	0.90	5.79E-10
Ciliary transition zone	0.90	5.79E-10
Photoreceptor outer segment membrane	0.90	5.79E-10
Inhibitory synapse	0.90	5.79E-10
Ciliary membrane	0.90	5.79E-10
Ciliary plasm	0.90	5.79E-10
9plus0 non motile cilium	0.90	5.79E-10
Voltage gated sodium channel complex	0.87	8.25E-09
Photoreceptor inner segment	0.87	8.25E-09
Axonemal dynein complex	0.87	8.25E-09
Cation channel complex	0.87	8.25E-09
Potassium channel complex	0.87	8.25E-09
Sodium channel complex	0.87	8.25E-09
Sperm midpiece	0.87	8.25E-09
Non motile cilium	0.87	8.25E-09
Photoreceptor cell cilium	0.87	8.25E-09

Table S6 Molecules, IC50, and working concentrations for the CSC targeting experiments.

Molecule Name	IDs	MCF7 concentrations	MDA-MB-231 concentrations
triptolide	BRD-K81258678<br>Sigma (cat. # S-645900)	IC50 = 6.375 nM<br>10nM; 20nM	IC50 = 4.915 nM<br>10nM; 50nM
OTS-167	BRD-K53417444<br>Vinci-Biochem (cat. # CAY-16873-5)	IC50 = 52.62 nM<br>50nM; 100nM	IC50 = 29.67 nM<br>50nM; 100nM
granisetron	BRD-A10967948<br>DBA (cat. # SC-203983)	IC50 = 1173 μM<br>300μM; 500μM	IC50 = 740.6 μM<br>300μM; 500μM
A-443654	BRD-K88573743<br>DBA (cat. # HY-10425)	IC50 = 26,91 nM<br>50nM; 100nM	IC50 = 302.5 nM<br>50nM; 100nM
leflunomide	BRD-K78692225<br>DBA (cat. # HY-B0083)	IC50 = Undetermined<br>100μM; 200μM	IC50 = 146.9 μM<br>25μM; 50μM
quinacrine	BRD-A45889380<br>DBA (cat. # HY-13735A)	IC50 = 1.791 μM<br>500nM; 5μM	IC50 = 1.787 μM<br>500nM; 5μM

Table S7 The Running time and memory footprint of DREDDA and other methods for comparison. Each method is tested on a workstation with Intel® Xeon® Gold 6238R CPUs using 112 logical processors or one NVIDIA® V100 GPU.

Method	Running Time	Memory requirement
DREDDA	Training: 286.3s ± 5.2s<br>Inference: 34.6s ± 1.2s	Training: 1.2GB<br>Inference: 1.2GB
GEP + Cosine Similarity	Inference: 0.50s ± 0.12s	minimal additional memory other than original data
PA + Cosine Similarity	PA computation: 520.0s ± 4.8s<br>Inference: 0.097s ± 0.01s	PA computation: 4.0GB<br>Inference: minimal additional memory other than original data
GEP + Jaccard Similarity	Inference: 62.0s ± 2.3s	minimal additional memory other than original data

Table S8 Detailed Network Architecture and Training Configurations of DREDDA.

Network Module / Training Configurations	Selected Hyperparameter	Range of Hyperparameter Selection
Source domain encoder	Input dim: (1000)	Top [500, **1000**, and 2000] genes with highest MI values were tried out
	Fully Connected Layer: (1000) >> (100)	Size of the intermediate layer: [50,**100**,200]
	Fully Connected Layer: (100) >> (50)	Size of the bottleneck layer: [**50**,100,200]
Target domain encoder	Input dim: (1000)	Top [500, **1000**, and 2000] genes with highest MI values were tried out
	Fully Connected Layer: (1000) >> (100)	Size of the intermediate layer: [50,**100**,200]
	Fully Connected Layer: (100) >> (50)	Size of the bottleneck layer: [**50**,100,200]
Shared decoder	Fully Connected Layer: (50) >> (100)	Size of the intermediate layer: [50,**100**,200]
	Fully Connected Layer: (100) >> (1000)	
Task classifier	Fully Connected Layer: (1000) >> (100)	Size of the intermediate layer: [50,**100**,200]
	Fully Connected Layer: (100) >> (80)	
	Fully Connected Layer: (80) >> (60)	Size of the adversarial features: [**60**,70,80]
	Fully Connected Layer: (60) >> (40)	
	Fully Connected Layer: (40) >> (20)	
	Task Output Layer: (20) >> (4)	

Adversarial domain classifier	Fully Connected Layer: (60) >> (40)	Size of the adversarial features: [**60**,70,80]
	Adversarial Output Layer: (40) >> (2)	
Learning rate (main network)	1e-4	Range of selection [1e-6,1e-5,**1e-4**,1e-3]
Learning rate (adversarial network)	1e-5	Range of selection [1e-6,**1e-5**,1e-4,1e-3]
Weight of adversarial training	1e-1	Range of selection [1e-4,1e-3,1e-2,**1e-1**]
Weight of domain confusion	1e-1	Range of selection [1e-4,1e-3,1e-2,**1e-1**]

REFERENCES

1. Ganin, Y. and Lempitsky, V. (2015). JMLR.org, pp. 1180-1189.
2. Tzeng, E., Hoffman, J., Zhang, N., Saenko, K. and Darrell, T. (2014) Deep Domain Confusion: Maximizing for Domain Invariance. *ArXiv*.
3. Borgwardt, K.M., Gretton, A., Rasch, M.J., Kriegel, H.-P., Schölkopf, B. and Smola, A.J. (2006) Integrating structured biological data by Kernel Maximum Mean Discrepancy. *Bioinformatics (Oxford, England)*, **22**, e49-57.
4. Paszke, A., Gross, S., Massa, F., Lerer, A., Bradbury, J., Chanan, G., Killeen, T., Lin, Z., Gimelshein, N., Antiga, L. *et al.* (2019). arXiv.
5. Napolitano, F., Sirci, F., Carrella, D. and di Bernardo, D. (2016) Drug-set enrichment analysis: a novel tool to investigate drug mode of action. *Bioinformatics*, **32**, 235-241.
6. Subramanian, A., Tamayo, P., Mootha, V.K., Mukherjee, S., Ebert, B.L., Gillette, M.A., Paulovich, A., Pomeroy, S.L., Golub, T.R. and Lander, E.S. (2005) Gene set enrichment analysis: a knowledge-based approach for interpreting genome-wide expression profiles. *Proceedings of the National Academy of Sciences*, **102**, 15545-15550.
7. Liberzon, A., Birger, C., Thorvaldsdóttir, H., Ghandi, M., Mesirov, J.P. and Tamayo, P. (2015) The Molecular Signatures Database (MSigDB) hallmark gene set collection. *Cell systems*, **1**, 417-425.
8. Napolitano, F., Carrella, D., Gao, X. and di Bernardo, D. (2020) gep2pep: a bioconductor package for the creation and analysis of pathway-based expression profiles. *Bioinformatics*, **36**, 1944-1945.
9. Szklarczyk, D., Santos, A., von Mering, C., Jensen, L.J., Bork, P. and Kuhn, M. (2016) STITCH 5: augmenting protein–chemical interaction networks with tissue and affinity data. *Nucleic Acids Research*, **44**, D380-D384.
